# Effect of an Online Video-Based Intervention to Increase HIV Testing in Men Who Have Sex with Men in Peru

**DOI:** 10.1371/journal.pone.0010448

**Published:** 2010-05-03

**Authors:** Magaly M. Blas, Isaac E. Alva, Cesar P. Carcamo, Robinson Cabello, Steven M. Goodreau, Ann M. Kimball, Ann E. Kurth

**Affiliations:** 1 Unit of Epidemiology, HIV and STD, School of Public Health and Administration, Universidad Peruana Cayetano Heredia, Lima, Peru; 2 NGO Via Libre, Lima, Peru; 3 Department of Epidemiology, School of Public Health and Community Medicine, University of Washington, Seattle, Washington, United States of America; 4 Department of Anthropology, University of Washington, Seattle, Washington, United States of America; 5 Departments of Global Health, and of Epidemiology, School of Public Health and Community Medicine, University of Washington, Seattle, Washington, United States of America; 6 School of Nursing, University of Washington, Seattle, Washington, United States of America; Tulane University, United States of America

## Abstract

**Background:**

Although many men who have sex with men (MSM) in Peru are unaware of their HIV status, they are frequent users of the Internet, and can be approached by that medium for promotion of HIV testing.

**Methods:**

We conducted an online randomized controlled trial to compare the effect of HIV-testing motivational videos versus standard public health text, both offered through a gay website. The videos were customized for two audiences based on self-identification: either gay or non-gay men. The outcomes evaluated were ‘intention to get tested’ and ‘HIV testing at the clinic.’

**Findings:**

In the non-gay identified group, 97 men were randomly assigned to the video-based intervention and 90 to the text-based intervention. Non-gay identified participants randomized to the video-based intervention were more likely to report their intention of getting tested for HIV within the next 30 days (62.5% vs. 15.4%, Relative Risk (RR): 2.77, 95% Confidence Interval (CI): 1.42–5.39). After a mean of 125.5 days of observation (range 42–209 days), 11 participants randomized to the video and none of the participants randomized to text attended our clinic requesting HIV testing (p = 0.001). In the gay-identified group, 142 men were randomized to the video-based intervention and 130 to the text-based intervention. Gay-identified participants randomized to the video were more likely to report intentions of getting an HIV test within 30 days, although not significantly (50% vs. 21.6%, RR: 1.54, 95% CI: 0.74–3.20). At the end of follow up, 8 participants who watched the video and 10 who read the text visited our clinic for HIV testing (Hazard Ratio: 1.07, 95% CI: 0.40–2.85).

**Conclusion:**

This study provides some evidence of the efficacy of a video-based online intervention in improving HIV testing among non-gay-identified MSM in Peru. This intervention may be adopted by institutions with websites oriented to motivate HIV testing among similar MSM populations.

**Trial registration:**

Clinicaltrials.gov NCT00751192

## Introduction

In Latin America in 2007, 58,000 people lost their lives due to AIDS, and approximately 100,000 were newly infected with HIV. An important risk factor in the epidemics of several countries of this region is unprotected sex between men who have sex with men (MSM). Countries with epidemics in this group include El Salvador, Guatemala, Honduras, Mexico, Nicaragua and Panama in Central America; and Bolivia, Chile, Ecuador and Peru in South America [1]. As a result, the potential impact of reducing risk in this population is significant.

In Peru, current interventions to target MSM are concentrated on peer education programs on streets, in bathhouses and in other places frequented by this population. Unfortunately this approach reaches only those MSM who are most easily identified (between 18% to 45% of the population), and neglects hidden MSM populations who are less likely to be gay-identified, such as closeted, younger and bisexual MSM [2]–[4].

In order to decrease HIV transmission among MSM, our efforts should be focused on finding new ways of promoting early identification and treatment of HIV and other Sexually Transmitted Infections (STI). A previous study conducted in Lima, the capital of Peru, in 2006 demonstrated that reaching high-risk MSM through the Internet is feasible [5]–[6]. The investigation showed that offering free HIV/syphilis tests as a compensation for participation without any additional payment increased the frequency of participation in an online survey. It also attracted high-risk MSM who were not tested for HIV, but were interested in a wide variety of preventive Web-based interventions [6], [7].

In general, Internet based interventions have proven to be an efficacious method of delivering health-related information to a large number of people who report high-risk sexual behaviours and who would otherwise not seek care or be targeted for health related interventions [8]. Several approaches, including the creation of educational sites, online risk reduction interventions, online partner notification, online counselling sessions and online STI testing, have met with some success [9]–[12]. Additionally, recent video-based offline and online interventions have proven to be effective in decreasing risk behaviors for HIV and in reducing STI acquisition among participants assigned to the intervention condition [13]–[14]. These factors, added to the importance of early identification of HIV disease, and the recent introduction of free Highly Active Antiretroviral therapy (HAART) in Peru, make it imperative to develop and test video-based online interventions to increase HIV testing among this high-risk population.

Given the need to triage public health resources in developing country settings, we proposed a randomized controlled trial (RCT) to study the association between video-based online interventions and proportions of HIV testing in gay-identified and non-gay-identified MSM.

## Methods

The protocol for this trial, supporting CONSORT checklist and a Spanish translation of the article are available as supporting information; see Protocol S1, Checklist S1 and Spanish S1.

From October 2007 to April 2008, we conducted a randomized controlled trial (RCT) to compare the efficacy of HIV-testing motivational videos versus standard public health text, offered through seven gay websites in Peru. Five of these were commercial gay websites (gayperu.com, peruesgay.com, diariodelimagay.com, deambiente.com, chicoslima.com) and two of them were advocacy gay websites (mhol.org.pe and runa.org.pe).

This study was conducted according to the principles expressed in the Declaration of Helsinki. The study protocol was approved by the Institutional Review Board of the University of Washington in Seattle and the non-governmental organization Vía Libre in Lima, Peru. All enrollees provided a web-based informed consent for the online questionnaire and a written informed consent for the collection of samples.

### Formative research

In order to develop the intervention, we conducted eight in-depth face-to-face interviews and two focus groups with each MSM subpopulation: gay and non-gay-identified MSM, and trans (transvestites: i.e., men who cross-dress; transgender and transsexual). During this period it became clear that a video depicting a story and a character to whom the audience can relate was more appealing to the participants than were characters animations. Additionally, the focus groups helped to identify the main reasons why participants don't get tested as well as the characters and the stories that should be depicted on the videos. We also identified the need for subtitles in the video; many MSM in Lima access the internet in semi-public cyber-cafes, so subtitles were needed both to facilitate privacy and because in some cybercafes audio was not available. Furthermore, in this period the need to customize the video according to self-identification: gay, non-gay and trans became clear. In a user-centered design approach [15], we involved members of the community, activists and researchers in the process of elaboration and postproduction editing of the video; and finally we pilot tested the video with the target population.

### Banner ads

We advertised animated banner ads that, if clicked, redirected the participants to our study website. The banner ads included the following message: ‘*SOMOS*’ (which was the name of the project and stands for optimal services for an opportune care), ‘*sex?*’, ‘*answer our anonymous survey’*, and ‘*click here*’. We did not include a reference about “HIV testing” in the banner. It is likely that this would have attracted participants only interested in being tested and therefore lowered the power to detect a difference between both arms of the intervention.

### Study website

Our website included a link to the online survey, information about risk and benefits of participation, privacy policy information, frequently asked questions, and a phone number for the participants to call if they needed more information. The survey, designed using LimeSurvey [16], documented demographic characteristics, self-identified sexual orientation, sexual and non sexual risk behaviors for STIs, presence of STI symptoms, online sex-seeking behavior, history of previous HIV testing, reasons for not taking an HIV test, and participant's stage of change/readiness related to HIV testing. In the questionnaires, participants were not asked for any personally identifiable information. However, they were asked to enter their email address twice (they were allowed to give an anonymous address if preferred) that was used to link the profile of each participant who attended to the clinic with his intervention arm assignment.

#### Eligibility Criteria

In order to be randomized to one of the interventions, the participants had to fulfill all of the following eligibility criteria: (1) be 18 years of age or older, (2) be a man and report having had sex with men, (3) be a resident of Lima, Peru, (4) answer the survey from Lima, Peru (5) not have been tested for HIV during the last year, (5) have an email address that when typed twice matched and, (6) do not report being HIV positive.

In order to avoid self-missrepresentation we did not make eligibility criteria transparent. Also, we allowed persons who may have only been interested in knowing more about the survey to view it without participating. Finally, we did not provide financial incentives for survey completion, in order to reduce participation or volunteer bias. After the participant finished the baseline risk assessment and entered his email address twice, our system assessed the fulfillment of all the eligibility criteria. The participant was asked to participate in the intervention only if he met these criteria.

### Types of interventions

Once the participant agreed to participate in the intervention and fulfilled all the eligibility criteria, he was randomly presented with either a video or a text. The randomization was simple, computer-based and was automatically done by an algorithm that evaluated each case and used a random number generator to make an independent assignment.

The videos had a length of five minutes and were customized for three audiences based on self-identification: non-gay (Video S1), gay (Video S2), and trans (Video S3) [17]–[19]. We report in this paper only on the first two audiences because we lacked power to evaluate the third (sample size = 21). The video targeted to gay-identified MSM was presented if the participant self identified as “gay” or “caleta” (men who are closeted or semi-closeted). The video targeted to non-gay-identified MSM was presented if the participant self-identification was heterosexual, bisexual, or “flete” (young male prostitutes). The control condition text was obtained from a current intervention to increase HIV testing in Mexico [20], since there was no existing intervention to increase HIV testing through the Internet in Peru.

The videos were framed within the health-belief model, which focuses on the attitudes and beliefs of individuals, and within the stages of change theory, which shows that people tend to progress through different stages on their way to successful behavioral change [21], [22]. The videos incorporated ways to overcome the following different reasons why MSM don't get tested for HIV: (1) fear of the consequences of a positive test result, (2) feeling that they have never been at risk for infection, (3) fear of discrimination, (4) fear of lack of confidentiality of health care personnel, (5) fear of not getting support from family/friends/partner, (6) lack of knowledge of where to get tested, (7) not being able to pay for the HIV test, (8) not being able to pay for the HIV treatment.

The videos transitioned through the stages of change of precontemplation, contemplation, preparation for getting tested and action (the actual testing). Both, the video and the text motivated the participants to visit the clinic to get tested for HIV for free.

### Evaluation of Outcome measures

The stages of change theory was used to classify the participants' stage of positive behavior of getting an HIV test [22]. If the participants reported that they were not planning to take the test within the next 6 months, they were classified in the precontemplation stage. If they intended to take the test within the next 6 months, they were in the contemplation stage. If they indicated that they were planning to take the test within the next 30 days they were classified as being in the preparation stage. Participants who took the test within the last year were categorized in the action stage, and participants who got tested at least once per year were classified in the maintenance stage. Participants in the action and maintenance stage were not randomized to any of the interventions.

The outcomes evaluated were changes from one stage to the next one: from precontemplation to contemplation, from contemplation to preparation and from any stage to action.

### Statistical Analysis

To calculate our sample size we assumed that a video-based intervention compared to a text-based intervention would increase the proportion of HIV testing by 12%. Assuming a proportion of HIV testing of 3.0% in our control intervention and 15.0% in our active intervention, our estimated sample size was 87 participants for each arm of the intervention. This will provide an 80% power at the 95% significance level to see an absolute difference of 12% in testing proportions between the two arms. Condition assignments were masked for investigators.

In order to evaluate the effect of the interventions, we compared the proportion of participants tested for HIV from each of the study arms using an intent-to-treat analysis. The email addresses that participants self-reported on the online survey were matched with those self-reported at the study site to allow the identification of the experimental condition to which participants were randomized. To test the hypothesis that the video-based intervention was more effective than the text-based intervention we used Mantel Haenszel adjusted Relative Risks. We tested differences in the proportion of participants who (i) planned to get tested for HIV within the next 6 months, (ii) within the next 30 days, (iii) who made an Internet appointment to the clinic and (iv) who attended the clinic.

To test the hypothesis that the video-based intervention compared to the text-based intervention increased the likelihood of HIV testing at the clinic in a survival model, we calculated adjusted Hazard Ratios using the Cox proportional hazards model. The analysis was performed in Stata 8.0.

As the intervention and randomization were different for gay and non-gay identified populations, the analysis was conducted independently for each of the two trials.

## Results

**Figure 1:**
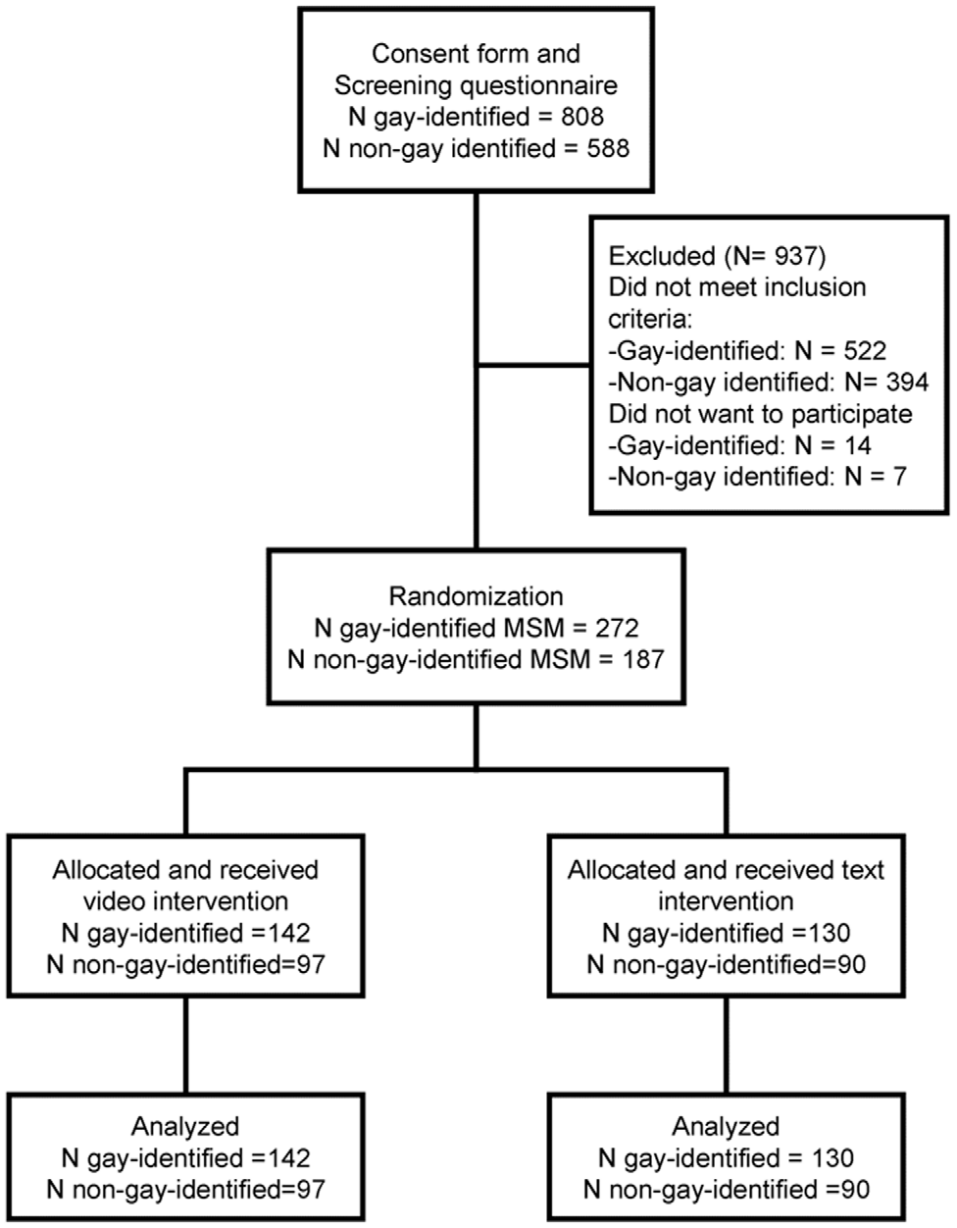
Randomization process.

We received 1481 surveys. Of these, 808 were from participants who were gay-identified, 588 from participants who were non-gay identified, 21 from trans and the reminder were from women and heterosexual males (Figure 1). We report only results from the gay and non-gay identified MSM group.

### Participant characteristics

In the non-gay identified group, 97 men were randomly assigned to the video-based intervention and 90 to the text-based intervention. In the gay-identified group, 142 men were randomized to the video-based intervention and 130 to the text-based intervention (Figure 1). There were no significant differences in demographic characteristics between participants of the video-based intervention and the text-based intervention in the non-gay-identified MSM group. However, in the gay-identified MSM group, participants from the video-based intervention were older than participants from the text-based intervention (p = 0.04; Table 1).

**Table 1:** Comparison of demographic characteristics, access to HIV information, risk behaviors for STI and STI symptoms by intervention group in the non-gay and gay-identified MSM group.

	Non-gay-identified MSM			Gay-identified MSM		
	Video	Text	P	Video	Text	P
	N(%)	N(%)		N(%)	N(%)	
**Mean age (range)**	26.4 (18–50)	26.2 (18–54)	0.84	26.9 (18–52)	25.0 (18–61)	0.04
**Education**			0.45			0.38
<High school	6 (6.3)	2 (2.2)		3 (2.1)	5 (3.9)	
High school graduate	13 (13.5)	13 (14.4)		18 (12.8)	22 (17.3)	
University/Technical non-graduate	33 (34.4)	38 (42.2)		55 (39.0)	53 (41.7)	
University/Technical graduate	44 (45.8)	37 (41.1)		65 (46.1)	47 (37.0)	
**Place of Internet access**			0.41			0.53
Commercial cybercafé	48 (50.5)	40 (44.4)		60 (43.2)	50 (39.4)	
Home/Work	47 (49.5)	50 (55.6)		79 (56.8)	77 (60.6)	
**Sexual orientation**			0.19			0.05
Homosexual	21 (21.6)	27 (30.0)		130 (91.5)	127 (97.7)	
Bisexual	76 (78.4)	63 (70.0)		12 (8.5)	3 (2.3)	
**Self-identification**			0.25			0.72
Bisexual	74 (76.3)	78 (86.7)		—	—	
Heterosexual	8 (8.3)	6 (6.7)		—	—	
Man	13 (13.4)	6 (6.7)		—	—	
Flete (Sex worker)	2 (2.1)	0 (0.0)		—	—	
Gay	—	—		123 (86.6)	115 (88.5)	
Caleta	—	—		19 (13.4)	15 (11.5)	
**Sexual role**			0.45			0.48
Insertive	38 (39.6)	27 (31.0)		11 (7.9)	16 (12.5)	
Receptive	10 (10.4)	12 (13.8)		45 (32.4)	39 (30.5)	
Versatile	48 (50)	48 (55.2)		83 (59.7)	73 (57.0)	
**Received information about HIV last year**	31 (33)	33 (37.1)	0.64	54 (39.1)	48 (37.8)	0.90
**Used condom at last intercourse**	47 (50.0)	44 (53.0)	0.88	60 (43.5)	64 (52.9)	0.26
**STI symptoms**						
Disuria	18 (18.6)	17 (18.9)	1.00	19 (13.4)	16 (12.3)	0.86
Uretral discharge	6 (6.2)	7 (7.8)	0.78	8 (5.6)	8 (6.2)	1.00
Anal discharge	1 (1)	1 (1.1)	1.00	0 (0.0)	2 (1.5)	0.23
Genital ulcers in penis	5 (5.2)	4 (4.4)	1.00	8 (5.6)	8 (6.2)	1.00
Genital ulcers in anus	7 (7.2)	4 (4.4)	0.54	4 (2.8)	9 (6.9)	0.16
Warts in penis	4 (4.1)	0 (0)	0.12	6 (4.2)	1 (0.8)	0.12
Warts in anus	5 (5.2)	2 (2.2)	0.45	8 (5.6)	5 (3.9)	0.58

There were also no differences regarding sexual orientation, self-identification, sexual role, and HIV-related information received during the last year between both arms of the intervention in the non-gay-identified MSM group. However, in the gay-identified MSM group, participants from the video-based intervention reported a higher percentage of bisexuality than participants in the text-based intervention (p = 0.05; Table 1). No statistically significant difference was found regarding condom use at last sexual intercourse, presence of STI symptoms during the last year, previous HIV test, and frequency of HIV testing between the arms of the intervention in either the gay and non-gay-identified MSM group (Table 1 and 2). Furthermore, before being exposed to the intervention, there were no significant differences regarding planning to getting tested for HIV within the next 6 months or 30 days between both arms of the intervention in either the gay and not gay-identified MSM group (Table 2).

**Table 2:** Comparison of history of HIV testing and plans for getting tested by intervention group in the non-gay and gay-identified MSM group.

	Non-gay-identified MSM			Gay-identified MSM		
	Video	Text	P	Video	Text	P
	N(%)	N(%)		N(%)	N(%)	
**Tested for HIV before** *			0.63			0.24
Never	73 (75.3)	62 (68.9)		76 (53.5)	83 (63.9)	
1–2 years ago	15 (15.5)	14 (15.6)		38 (26.8)	30 (23.1)	
2–5 years ago	5 (5.2)	8 (8.9)		23 (16.2)	12 (9.2)	
More than 5 years ago	4 (4.1)	6 (6.7)		5 (3.5)	5 (3.9)	
**Frequency of HIV testing**			0.82			0.80
Only once	14 (66.7)	19 (76.0)		29 (48.3)	28 (62.2)	
Twice a year				1 (1.7)	0 (0.0)	
Every year	2 (9.5)	3 (12.0)		14 (23.3)	8 (17.8)	
Every 2–3 years	3 (14.3)	2 (8.0)		11 (18.3)	7 (15.6)	
Every 4–5 years	0 (0.0)	0 (0.0)		3 (5.0)	1 (2.2)	
Other	2 (9.5)	1 (4.0)		2 (3.3)	1 (2.2)	
**Planning to get tested for HIV in the next 6 months (before being exposed to the intervention)**	60 (66.0)	54 (63.5)	0.76	96 (73.9)	86 (71.7)	0.78
**Planning to get tested for HIV in the next 30 days (before being exposed to the intervention)**	32 (36.4)	19 (24.1)	0.10	48 (38.1)	38 (33.9)	0.59

*Respondents tested within 12 months (in the action stage) were not randomized.

When comparing HIV testing history between non-gay and gay identified participants, 135 (72.2%) of non-gay identified participants and 159 (58.5%) of gay-identified participants reported they have never tested for HIV before (p = 0.004).

### Intervention effects

After a mean of 125.5 days of observation (range 42–209 days), 18 gay-identified MSM and 11 non-gay identified MSM attended our clinic to get tested for VIH. There was not a significant difference in the reporting of intentions of getting tested for HIV within the next 6 months among participants from the video-based intervention and the text-based intervention in both groups, gay (RR = 1.75; 95% Confidence Interval (CI): 0.77–3.97) and non-gay (RR = 1.43; 95% CI: 0.87–2.36) identified MSM. There was also not a significant difference in the reporting of intentions of getting tested for HIV within the next 30 days (RR = 1.54; 95% CI: 0.74–3.20), in making an Internet appointment (RR = 1.11; 95% CI: 0.88–1.39) and in attending the clinic for HIV testing (RR = 1.07; 95% CI: 0.40–2.85) among participants from the video-based intervention and the text-based intervention in the gay identified MSM group. However, participants of the video-based intervention from the non-gay identified MSM group were more likely to report intentions of getting tested for HIV within the next 30 days (RR = 2.77; 95% CI: 1.42–5.39), and also were more likely to make an Internet appointment (RR = 1.48; 95% CI: 1.13–1.95) and to attend the clinic for testing (11.3% vs. 0%; p = 0.001). compared to participants from the text-based intervention (Table 3 and 4).

**Table 3:** Intervention effects in the non-gay identified MSM group.

	Assignment		
	Video	Text	
	N (%)	N (%)	Risk Ratio^c^ (95% CI)
**Planning to get tested for HIV in the next 6 months after being exposed to the intervention** a			
Yes	18 (62.1)	13 (43.3)	1.43 (0.87–2.36)
No	11 (37.9)	17 (56.7)	Ref
**Planning to get tested for HIV in the next 30 days after being exposed to the intervention** b			
Yes	15 (62.5)	4 (15.4)	2.77 (1.42–5.39)
No	9 (37.5)	22 (84.6)	Ref
**Made an Internet appointment**			
Yes	64 (66.0)	40 (44.4)	1.48 (1.13–1.95)
No	33 (34.0)	50 (55.6)	
**Attended to the clinic**			
Yes	11 (11.3)	0 (0.0)	…^d^
No	86 (88.7)	90 (100)	Ref

a Restricted to those not planning to getting tested for HIV within the next 6 months prior to being exposed to the intervention.

b Restricted to those who were planning to getting tested for HIV withing the next 6 months but who were not planning to getting tested within the next 30 days prior to being exposed to the intervention.

c Risk Ratio of planning to get tested for HIV in the next 6 months and the next 30 days was calculated using Mantel Haenszel and the Risk Ratio for attending the clinic was calculated using Cox proportional hazards regression.

d Hazard Ratio cannot be estimated.

**Table 4:** Intervention effects in the gay-identified MSM group.

	Assignment			
	Video	Text	Risk Ratio^c^ (95% CI)	
	N (%)	N (%)	Crude (95% CI)	Adjusted (95% CI)^d^
**Planning to get tested for HIV in the next 6 months after being exposed to the intervention** a				
Yes	17 (58.6)	11 (35.5)	1.65 (0.94–2.91)	1.75 (0.77–3.97)
No	12 (41.4)	20 (64.5)		
**Planning to get tested for HIV in the next 30 days after being exposed to the intervention** b				
Yes	19 (50.0)	8 (21.6)	2.31 (1.16–4.61)	1.54 (0.74–3.20)
No	19 (50.0)	29 (78.4)		
**Made an Internet appointment**				
Yes	84 (59.2)	71 (54.6)	1.08 (0.88–1.33)	1.11 (0.88–1.39)
No	58 (40.9)	59 (45.4)		
**Attended to the clinic**				
Yes	8 (5.6)	10 (7.7)	1.03 (0.39–2.70)	1.07 (0.40–2.85)
No	134 (94.4)	120 (92.3)		

a Restricted to those not planning to getting tested for HIV within the next 6 months prior to being exposed to the intervention.

b Restricted to those who were planning to getting tested for HIV withing the next 6 months but who were not planning to getting tested within the next 30 days prior to being exposed to the intervention.

c Risk Ratio of planning to get tested for HIV in the next 6 months and the next 30 days was calculated using Mantel Haenszel and the Risk Ratio for attending the clinic was calculated using Cox proportional hazards regression.

d Adjusted for age and sexual orientation.

**Figure 2:**
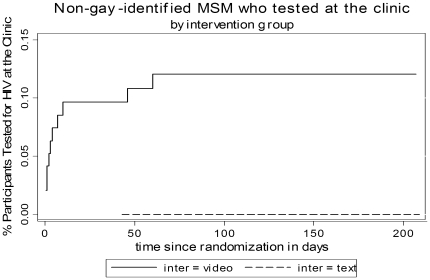
Percentage of non-gay-identified MSM tested for HIV by time since randomization (in days).

**Figure 3:**
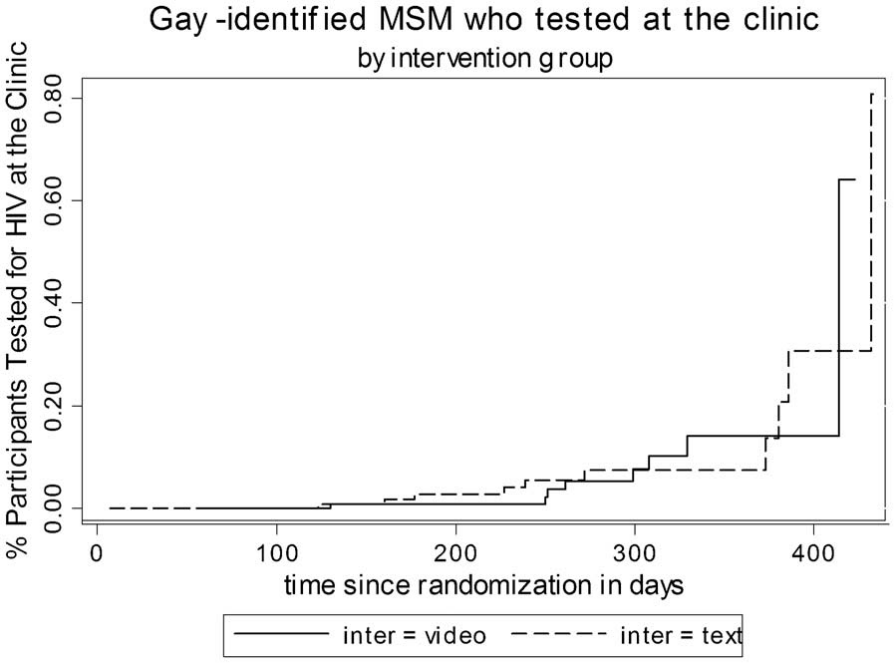
Percentage of gay-identified MSM tested for HIV by time since randomization (in days).

The median number of days and range of attendance to the clinic among non-gay identified MSM from the video-based intervention who attended to our site was 3 days (0.5, 60 days). The median number of days and range of attendance to the clinic in the video and text-based intervention among gay-identified MSM who attended our site was 280 days (130–414) and 255.5 days (123–432) respectively (Figure 2 and 3). Among the non-gay identified MSM group who attended the clinic, 8 out of 11 men have never been tested for HIV. In the gay-identified MSM group 10 out of 18 men who attended our clinic never tested for HIV before.

### Intervention acceptability

In the non-gay-identified MSM group, the acceptability of the intervention was similar among participants of the video-based intervention and the text-based intervention (p = 0.58). In the gay-identified MSM group, the acceptability of the video was higher than the acceptability of the text (p = 0.002; Table 5).

**Table 5:** Evaluation of the interventions and services that participants from the gay and non-gay identified MSM group were interested in receiving at the clinic.

	Non-gay-identified MSM			Gay-identified MSM		
	Video	Text	P	Video	Text	P
	N(%)	N(%)		N(%)	N(%)	
**Evaluation of the intervention**			0.58			0.002
Very good	55 (60.0)	44 (50.0)		93 (66.9)	61 (47.7)	
Good	30 (32.6)	34 (38.6)		39 (28.1)	45 (35.2)	
Regular	6 (6.5)	9 (10.2)		7 (5.0)	15 (11.7)	
Bad	0	0		0	5 (3.9)	
Very bad	1 (1.1)	1 (1.1)		0	2 (1.6)	
**Services participants were interested in receiving at the clinic**						
Medical care	56 (57.7)	34 (37.8)	0.008	84 (59.2)	65 (50.0)	0.14
Counseling	58 (59.8)	32 (35.6)	0.001	77 (54.2)	63 (48.5)	0.40
Condoms and lubricants	48 (49.5)	31 (34.4)	0.04	70 (49.3)	61 (46.9)	0.72
Rapid HIV test	63 (65.0)	42 (46.7)	0.01	94 (66.2)	60 (46.2)	0.001
ELISA for HIV	37 (38.1)	27 (30.0)	0.28	39 (27.5)	43 (33.1)	0.36
Syphilis test	42 (43.3)	25 (27.8)	0.03	55 (38.7)	45 (34.6)	0.53

### Services participants are interested in receiving at the clinic

Participants in the video-based intervention among the non-gay identified MSM group were more interested in receiving the following for free at the clinic compared to those in the text based intervention: medical care, counseling, condoms and lubricants, rapid HIV testing and syphilis tests. Participants in the video-based intervention among the gay identified MSM group were more interested in the rapid HIV test compared to participants from the text-based intervention (Table 5).

## Discussion

The goal of this study was to examine the effectiveness of an online video-based intervention in increasing the willingness to take an HIV test in the next 6 months and next 30 days after being exposed to the intervention and in increasing actual HIV testing in gay-identified and non-gay-identified MSM in Peru. This last goal was very challenging considering that the target population to which our intervention was oriented is a marginalized population in Peru that lives in an environment where being MSM and having HIV is highly stigmatized and where the availability of free HAART is not always known. Additionally, it is important to consider that we were targeting MSM who have not been tested for HIV during the last year, a population more reluctant to get tested [5]. Furthermore, we provided neither monetary compensation for participation nor additional resources to cover the cost of transportation to our site.

Neither video (either targeted to gay and non-gay identified MSM) increased the percentage of participants planning to get tested for HIV within the next 6 months; thus we were not able to produce a measureable move in the participants from a precontemplation stage to a contemplation stage. The precontemplation stage has been found to be a stable stage. In a study of HIV risk in women, participants' stage of change with condom use was assessed two different times one year apart, and more than 50% of the participants remained in that stage one year later [23]. According to a study conducted by Prochaska *et al.*, in the precontemplation stage participants believe the cons of changing the problem behavior are higher than the pros, and the progress from precontemplation to contemplation involves an increase in the evaluation of the pros of changing [19]. Although both of our videos highlighted these advantages, the lack of change observed could have been due to the fact that 1) the intervention was not tailored to specific fears or reasons each participant had for not getting tested but to a group of reasons for not getting tested identified in the formative research, 2) to the short period of the intervention (the length of the videos were five minutes), or 3) the short time period from the end of the intervention to the evaluation (minutes).

The video targeted to non-gay identified MSM significantly increased the percentage of participants planning to get tested for HIV within the next 30 days. This result illustrates that we were able to produce a change from contemplation to preparation, and also increase the percentage of participants who made an Internet appointment and who finally got tested at our clinic (a change to action). The median time to testing among those who came to the clinic was only 3 days However, the video targeted to gay identified MSM did not produce such effects. In fact, for them the median time to testing among those who attended our site was long (280 days). It is likely that some other reason such as a recent risky behaviour triggered the visit for HIV testing.

There may be several explanations for why the video targeted to non-gay identified MSM produced significant advances in the stages of change. A reason may be that the non-gay identified MSM group was newer to HIV testing in general than the gay-identified MSM group (72.2% of non-gay identified participants versus 58.5% of gay-identified participants had never tested for HIV before), so they were more ready for a little information to motivate them towards testing, whereas the gay-identified MSM group already have been through the rational pros and cons of getting tested, so the entire intervention-either text or video-added nothing new to them. Additionally, the video targeted to non-gay identified MSM did not show a person testing positive as in the video targeted to gay identified MSM. This may have made the possibility of testing positive less evident, which is recognized as the most common reason why MSM don't want to get tested for HIV [24], [25]. It could also be due to the fact that the message from the text-based intervention did not work as well as the video in improving changes in the non-gay identified MSM group but it worked well for the gay identified group. Finally, there is the possibility that the non-gay identified MSM group have a stronger influence by videos than text compared to the gay identified MSM population.

In gay-identified and non-gay identified MSM, the acceptability of the video and text was high (more than 80% rated these interventions as ‘very good’ or ‘good’). In the non-gay identified group the participants who saw the video were more interested than the participants who saw the text in receiving services such as free medical care, counseling, condoms and lubricants, rapid HIV testing, and syphilis testing at the clinic. In contrast, in the gay-identified group, the participants from the video-based intervention were more interested in the rapid HIV tests only compared to participants from the text-based intervention. This implies that the message obtained from the video in the gay-identified MSM group was focused on the HIV test while in the non-gay identified MSM group was more diversified to a variety of health care options. This finding can also explain why a significantly higher number of non-gay identified participants from the video-based intervention compared to the text-based intervention attended the clinic.

Empirical data in either education or psychological literature suggest that effective learning can be improved by multimodal presentations that combines auditory-verbal and visual presentations [26]. Additionally, videos compared to text are a visual medium that can depict characters to whom patients can relate and also they can provide information and model successful strategies for overcoming barriers to risk reduction [27], [28].

To our knowledge this is the first RCT addressing HIV and STI issues on the Internet in a developing country setting. Previous RCTs, such as the evaluation of a home-based computer network in reducing social isolation in people living with AIDS [29]; the evaluation of an online reminder system using pagers for improving antiretroviral adherence [30]; the evaluation of the effect of online tailored and untailored interventions to increase condom use, STI testing and HIV testing in MSM [31]; and the evaluation of the acceptability and efficacy of Internet-delivered HIV risk reduction interventions among rural MSM [10]; have been conducted in developed-country settings where means of accessing the Internet (more at home than at commercial cybercafes) and the speed of the connection are different [7]. The feasibility of conducting this online RCT in a resource-constrained setting and the use of actual HIV testing as a measure of effectiveness of the intervention opens new possibilities not only to further explore and refine new interventions but to extend those to other populations and diseases.

An interesting finding was the high number of participants who made an Internet appointment and who finally did not come. Some possible explanations for this are that the Internet is attractive to MSM due to the anonymity that it provides and will work mainly when participants are online but not if they are required to appear in person. Accesibility to the clinic may be another issue. Lima is a 33 miles long city with 8 million population that commutes mainly through slow public transportation, making it hard for potential participants to reach our only clinic located at dowtown Lima. Furthermore, we did not include reminders of appointment, since this study was designed to assess specifically the effect of a video over a text. In future studies the implementation of follow-up messages that reinforce interventions, and personalized reminders, may increase the number of participants who finally attend the clinic. Additionally, we developed videos of 5 minutes of duration that were intended to address collectively all the reasons for not getting tested for each group; this video may not have been tailored enough to specific fears that individual participants may have had.

Our study has some limitations. First, our sample is not representative of the MSM population from Lima or Peru, and also may not represent the entire online population of MSM who visit all available Peruvian gay websites. We attempted to reach those at highest risk with Internet access. Second, our sampling is likely to be biased in terms of educational background and age. Third, we were unable to collect data about participants who may have attended other clinics to receive testing. Fourth, we may have self-misrepresentation of some participants leading to misclassification (e.g., female participants answering as a male), which may decrease the internal validity of our study. Finally, we were unable to measure compliance with the interventions (i.e., if participants read the text or saw the video).

The Internet has emerged as an alternative method that can be used to engage high-risk populations from resource constrained settings in HIV preventive interventions oriented to provide earlier diagnosis and treatment for HIV. Future online interventions in Peru should target specific subpopulations among the gay-identified and non-gay identified MSM groups and should be tailored specifically to the most important reasons the participants have for not getting tested for HIV. Follow-up should also take into account the appropriate length of time for participants' likely movement through the stages of change, and should also include a wide variety of HIV testing sites where participants can attend.

## Supporting Information

Protocol S1 Study protocol
(0.21 MB DOC)

Checklist S1 CONSORT checklist
(0.05 MB DOC)

Spanish S1 Spanish translation of the article
(0.25 MB DOC)

Video S1 For non-gay identified MSM. Video oriented to motivate the non-gay identified men who have sex with men population to get tested for HIV
(9.62 MB AVI)

Video S2 For gay-identified MSM. Video oriented to motivate the gay-identified population to get tested for HIV
(10.01 MB AVI)

Video S3 For trans. Video oriented to motivate the trans population to get tested for HIV
(23.23 MB AVI)

## References

[1] UNAIDS (2007). *AIDS Epidemic Update*.

[2] Orellana ER, Picciano JF, Roffman RA, Swanson F, Kalichman SC (2006). Correlates of nonparticipation in an HIV prevention program for MSM.. AIDS Educ Prev.

[3] Kusunoki L, Guanira J, Navarro C, Velasquez C (2005). Report of Monitoring the declaration of commitment on HIV/AIDS 2005.. http://data.unaids.org/pub/Report/2006/2006_country_progress_report_peru_en.pdf.

[4] Peru Country Progress Report UNGASS (2008). http://data.unaids.org/pub/Report/2008/peru_2008_country_progress_report_sp_es.pdf.

[5] Blas M (2007). *Web-based survey to assess risk behaviors for sexually transmitted infections and HIV among men who have sex with men from Peru*..

[6] Blas MM, Alva IE, Cabello R, Garcia PJ, Carcamo C (2007). Internet as a tool to access high-risk men who have sex with men from a resource-constrained setting: a study from Peru.. Sex Transm Infect.

[7] Curioso WH, Blas MM, Nodell B, Alva IE, Kurth AE (2007). Opportunities for providing web-based interventions to prevent sexually transmitted infections in Peru.. PLoS Med.

[8] Bull SS, McFarlane M, King D (2001). Barriers to STD/HIV prevention on the Internet.. Health Educ Res.

[9] Levine DK, Scott KC, Klausner JD (2005). Online syphilis testing–confidential and convenient.. Sex Transm Dis.

[10] Bowen AM, Horvath K, Williams ML (2007). A randomized control trial of Internet-delivered HIV prevention targeting rural MSM.. Health Educ Res.

[11] Centers for Disease Control and Prevention (CDC) (2004). Using the Internet for partner notification of sexually transmitted diseases. Los Angeles County, California, 2003.. MMWR Morb Mortal Wkly Rep.

[12] Ross MW (2002). The Internet as a medium for HIV prevention and counseling.. Focus.

[13] Warner L, Klausner JD, Rietmeijer CA, Malotte CK, O'Donnell L (2008). Effect of a brief video intervention on incident infection among patients attending sexually transmitted disease clinics.. PLoS Med.

[14] (2008). Online film successful in reducing certain HIV risk behaviors. At-risk people watch 9-minute video.. AIDS Alert.

[15] Nijland N, van Gemert-Pijnen J, Boer H, Steehouder MF, Seydel ER (2008). Evaluation of internet-based technology for supporting self-care: problems encountered by patients and caregivers when using self-care applications.. J Med Internet Res.

[16] LimeSurvey, the open source survey application.. http://www.limesurvey.org.

[17] Get tested with Via Libre (for non-gay identified MSM).. http://www.youtube.com/watch?v=mKeR9-YVswg.

[18] Get tested with Via Libre (for gay-identified MSM).. http://www.youtube.com/watch?v=QN4BmLopzG0.

[19] Get tested with Via Libre (for trans).. http://www.youtube.com/watch?v=nT131RlToeo.

[20] El Gremio, centro comunitario de Lucha Contra el SIDA. Campana Hazte la Prueba.. http://www.gremiosanluis.com/sexoseguro_prueba.htm.

[21] DiClemente CC, Prochaska JO, Fairhurst SK, Velicer WF, Velasquez MM (1991). The process of smoking cessation: an analysis of precontemplation, contemplation, and preparation stages of change.. J Consult Clin Psychol.

[22] Prochaska JO, Velicer WF, Rossi JS, Goldstein MG, Marcus BH (1994). Stages of change and decisional balance for 12 problem behaviors.. Health Psychol.

[23] Evers KE, Harlow LL, Redding CA, LaForge RG (1998). Longitudinal changes in stages of change for condom use in women.. Am J Health Promot.

[24] Kellerman SE, Lehman JS, Lansky A, Stevens MR, Hecht FM (2002). HIV testing within at-risk populations in the United States and the reasons for seeking or avoiding HIV testing.. J Acquir Immune Defic Syndr.

[25] Mikolajczak J, Hospers HJ, Kok G (2006). Reasons for not taking an HIV-test among untested men who have sex with men: an Internet study.. AIDS Behav.

[26] Mayer RE (2001). *Multimedia Learning*.

[27] Myint-U A, Bull S, Greenwood GL, Patterson J, Rietmeijer CA (2008). Safe in the City: Developing an Effective Video-Based Intervention for STD Clinic Waiting Rooms.. Health Promot Pract.

[28] O'Donnell L, San Doval A, Duran R, O'Donnell CR (1995). The effectiveness of video-based interventions in promoting condom acquisition among STD clinic patients.. Sex Transm Dis.

[29] Flatley-Brennan P (1998). Computer network home care demonstration: a randomized trial in persons living with AIDS.. Comput Biol Med.

[30] Safren SA, Hendriksen ES, Desousa N, Boswell SL, Mayer KH (2003). Use of an on-line pager system to increase adherence to antiretroviral medications.. AIDS Care.

[31] Bull SS, Lloyd L, Rietmeijer C, McFarlane M (2004). Recruitment and retention of an online sample for an HIV prevention intervention targeting men who have sex with men: the Smart Sex Quest Project.. AIDS Care.

