# Supplementary material for: Effect of an Online Video-Based Intervention to Increase HIV Testing in Men Who Have Sex with Men in Peru
Source: PLoS One. 2010 May 3;5(5):e10448. doi: 10.1371/journal.pone.0010448 (PMC2862715; doi:10.1371/journal.pone.0010448)
Supplement: Protocol S1 — Study protocol (0.21 MB DOC) [file pone.0010448.s001.doc]

PROTOCOL

**Web-based randomized controlled trial to increase HIV testing in men who have sex with men in Lima- Peru**

**Universidad Peruana Cayetano Heredia**

**Magaly M. Blas**

**Cesar Carcamo**

**Isaac E. Alva**

**Via Libre**

**Robinson Cabello**

**University of Washington**

**Ann E. Kurth**

**Steven Goodreau**

**Ann Marie Kimball**

**March 20, 2007**

**I. ABSTRACT**

Men who have sex with men (MSM) account for the greatest burden of the HIV and Sexually Transmitted Infection (STI) epidemic in Peru. Current interventions that promote early identification and treatment of these diseases target a limited number of this population because they rely solely on peer education. To assess the use of the Internet as an alternative tool to reach this population we propose to deliver and compare an online video-based intervention and a standard public health text message to promote HIV testing in this marginalized population. The methodology we will use is divided in: (1) the development period, where we will select gay websites for banner advertisement, create and refine effective recruitment messages and design the baseline risk assessment and tailored and untailored interventions (2) the intervention period, where participants who meet the eligibility criteria will be randomized into the video or text intervention. The text intervention will be an untailored message with an invitation to a free HIV testing similar in content to other local websites; the video-based intervention will include tailored messages specific to the participant self-identification (3) in the outcome evaluation we will compare the proportion of HIV testing among MSM of each of the trial arms. If the Internet is an effective venue to reach MSM for HIV testing, health programs that target this population may start considering the delivery of Web-based interventions and other online prevention services to this underserved and hard-to-reach population.

**II. RESEARCH PLAN**

**A. SPECIFIC AIMS:**

Men who have sex with men (MSM) account for the greatest burden of the HIV epidemic in Peru and are an important bridge for HIV transmission into heterosexual women due to their high frequency of bisexuality.^1,2^ Thus, the population-attributable impact of reducing risk in this population is significant. To decrease HIV transmission among MSM our efforts should be oriented to promoting early identification and treatment of HIV and other Sexually Transmitted Infections (STI). Current interventions developed in Peru to target this population are based on peer education programs at streets, bathhouses and other places frequented by this population. This type of approach reaches only those MSM who are most easily identified (in the last sentinel surveillance only 18% MSM reported being approached by peer educators) and misses hidden MSM populations such as closeted, younger and bisexual MSM.^2^ For this reason new venues to access high-risk MSM difficult to target by traditional interventions should be identified. A previous Web-based survey developed by our group was able to reach through the Internet a core group of MSM not previously tested for HIV who had two important characteristics: 1) significantly higher risk behaviors for HIV/STI compared to MSM previously tested and 2) high frequency of self-reported STI symptoms.^3^ These factors, added to the proven effectiveness of HIV counseling and testing as an HIV prevention strategy and the recent introduction of free Highly Active Antiretroviral therapy (HAART) in Peru, make it imperative to develop and test online interventions to increase HIV testing among this high-risk population.^2^

Given the need to triage public health resources in developing settings we are proposing an effectiveness randomized controlled trial (RCT) to study the association between an online video-based intervention and proportions of HIV testing in an MSM population in Lima, Peru. Our hypothesis is that an active (tailored) intervention compared to a passive (untailored) intervention will produce an absolute increase of 10% in the proportion of HIV testing. The specific aims of our study are:

Aim 1: To develop an online video-based intervention targeted to MSM who have not been tested for HIV within the last year. This intervention will address the reasons MSM have for not getting tested and will be tailored to the participant self-identification: gay, non-gay or trans.

Aim 2: To conduct a RCT on the Internet. Participants will be randomized to either a video-based or a text-based intervention.

Aim 3: To compare the intentions of getting tested and the proportion of HIV testing among MSM randomized to the video-based and text-based intervention.

1. **BACKGROUND AND SIGNIFICANCE**

As of December 2005, an estimated 40.3 million [36.7-45.3] people worldwide are living with HIV, the highest level ever reached.^4^ In Peru, since the beginning of the epidemic in 1983 until June 2006, 18,508 cumulative AIDS cases and 24,454 cumulative HIV cases have been reported to the Peruvian Ministry of Health.^5^ Alarmingly, it is estimated that around 50,000 persons are still unaware of their HIV infection.^2^

The HIV epidemic in Peru is concentrated mainly among MSM, a group who has a national HIV prevalence of 13.9%. The prevalence of other STIs among this population is also high; 13.4% for syphilis and 46.3% for Herpes simplex virus type 2 (HSV-2).^6^ Among MSM, 26.5% report bisexuality and a low rate of consistent condom use especially with female partners (17% compared to 25.5% with male partners).^1^ This sexual behavior has increased the HIV transmission into heterosexual women, as indicated by the decreased HIV male/female ratio from 27/1 in 1987 to 2.95/1 in 2006.^5^ According to the last sentinel surveillance in MSM, only 18% have been approached by peer educators and only 42.5% have been tested for HIV before.^2^ Therefore, greater effort is needed to identify innovative, cost-effective interventions that successfully reach high-risk MSM.^3^ In this aspect, the Internet is a promising venue from which to target this population.

HIV disproportionately affects the young Peruvian population; in 2005 among the persons diagnosed with HIV 70% were between 20-39 years old. Seventy percent of the total HIV cases are concentrated in Lima, the capital of Peru. Compared to other cities in the country, Lima has the highest proportion of MSM who are HIV positive: 22.3%.^2,6^ Therefore, it constitutes a key place to deliver an intervention.

**Role of the Internet in the HIV epidemic**

The Internet constitutes a potential source of information in health care (more than 43% of Internet users have used the Internet to seek health information). Patients can search for information about their diagnosis, seek out health care providers, and explore treatment options.^7^ However, the Internet can also be used to solicit sexual partners.^8^ MSM is the population who more actively seek online sexual partners, at a rate approximately seven times more frequently than non-MSM.^9^ Seeking sexual intercourse on the Internet is a risk factor for HIV and STI transmission.^10-13^ Individuals who seek sex partners through the Internet may report a higher level of sexual risk behaviors such as higher number of partners, more anal sex, and more sexual exposure to partners known to be HIV positive compared to those who do not use the Internet to find sex partners.^10^

Studies developed with patients at STI clinics found also that among this core group, the Internet is a common venue for meeting and having sex with partners. In San Francisco, MSM with early syphilis infections reported the Internet as the most common place to meet sex partners, followed by bars, bathhouses and sex clubs.^14^ Having sexual intercourse with partners met online has also been directly linked to syphilis epidemics and HIV transmission, Klausner *et al.* in 2000 reported a syphilis outbreak among gay men linked to an online chat room in San Francisco^15^ and Tashima *et al.* in 2003 two cases of acute HIV infection acquired through meeting persons over the Internet.^16^

**Internet as a medium to deliver preventive interventions for HIV/STI**

Internet interventions have proven to be an efficacious method of delivering health-related information to a good number of people who report high-risk sexual behaviors and who would not otherwise seek care or be targeted for health related interventions.^9^ Several approaches, including the creation of educational sites, appealing banner advertisements with tailored feedback to the participants, online partner notification, online counselling sessions and online STI testing, have met with certain amounts of success.^17-19^ However, effective retention strategies of participants enrolled on the Internet need to be studied specially in longer follow-up studies. Bull *et al.* in 2004 lost 85% of participants to follow-up in a three-month online longitudinal study and Bowen *et al*. in 2006 lost 21% of participants in a one-week online RCT.^20,21^

Compared to non-computerized methods of data collection, Internet-based surveys have the advantages of automated randomization of persons and questions, automated branching, calculation of complex algorithms, consistency checks, automated data collection, and easy inclusion of graphics, video, audio and animation.^22^ Additionally, it is easier to identify patterns predictive of non-intentional and inconsistent responding, logical impossibilities, and inconsistencies which may increase the internal validity of our study. The Internet provides participants with the choice to access the survey at any time, which may increase the participation of people who may not respond through traditional approaches.^22^ Furthermore, given that most Internet approaches assure anonymity to the participants, they are less likely to be distorted in the direction of social desirability which may improve the sensitivity of our study when reporting HIV-risk related behaviors.^22^ Regarding data quality, Internet data collection yields very few missing data points and avoids data transcription errors because the information collected can be exported directly to a statistical software program.^22,23^

**Randomized control trials on the Internet**

Four previous RCT addressing HIV and STI have been conducted on the Internet. The first one evaluated the effect of a home-based computer network in reducing social isolation, improve confidence and skill in decision making among people living with AIDS (PLWA).^24^ The study found a reduction in social isolation, improvement in confidence but not skill in decision making in the intervention group. The second study tested the effect of a reminder system using pagers programmed with Web-based technology versus continue monitoring in improving antiretroviral adherence.^25^ The study found that the group who received the pagers had greater improvements in adherence than those who monitored their medications only. However, adherence in both groups was still poor (64% in the intervention group and 52% in the control group). The third one evaluated an online tailored and untailored intervention targeted to MSM.^26^ The intervention arm was framed within a stage of change context and encouraged the participants to consider incremental changes toward condom use with non-main partners, STI testing and HIV testing. Although the high attrition (complete follow-up data was available for only 15.2% of participants) did not allow an accurate interpretation of the results, the data showed that men in the intervention group were significantly more likely than men in the control group to indicate their willingness to go to an STI prevention website to obtain information on disease prevention. The fourth RCT was published recently and tested the acceptability and efficacy of an Internet-delivered HIV risk reduction intervention among rural MSM.^27^ At the end of the intervention HIV/AIDS knowledge, self-efficacy and outcome expectancies increased in the intervention group compared to the control group. These changes were maintained at one-week follow-up.

As we have seen, the Internet has emerged as an alternative, cost effective method to engage high-risk populations in prevention interventions oriented to reducing the incidence of HIV and STI and also in improving the antiretroviral adherence and quality of life of PLWA.^18^ Although numerous Internet-based interventions in developed countries have been developed and tested, little is known about their effectiveness in developing settings.

**Studies from developing settings**

The access to Internet in developing settings from the Americas is growing fast along with its use as a tool to provide HIV preventive educational material. ^28^ The use growth from 2000-2007 in Central America, South America and the Caribbean has been 624%, 327% and 704% respectively.^41^ Some examples of the use of the Internet for prevention are the creation of online educational sites oriented to improve the knowledge of local health-care providers about the HIV epidemic in Brazil, Russia and Vietnam,^29-31^ websites oriented to educate the youth about the risk factors for HIV acquisition and the different modes of protection in Mexico, Philippines, Nigeria and South Africa;^32-36^ online counseling sessions about sexual and reproductive health topics in the Philippines and the use of subsidized access to computers at commercial cybercafés as a mechanism to attract the young population in South Africa.^35,36^ The Internet has also been used to provide young gay men with messages about HIV prevention and a better knowledge about their rights in Guatemala^37^ and to build networks of MSM in areas with high discrimination against homosexuality, such as the Caribbean.^38^ In Brazil a website was developed to inform health and security staff of the penitentiary system about prevention and assistance programs related to HIV/AIDS ^39^ and, in China, an online survey was used to assess knowledge and sexual risk behaviors for HIV/STI among MSM recruited from gay venues.^40^

**Internet access in Peru**

Peru has one of the fastest expanding Internet networks in the world. The growth in Internet usage from 2000-2007 has been 82.8% with an estimated number of ten million Internet users by January of 2006.^41^ The phenomenon that has

facilitated the increasing access to the Internet is the dissemination of ‘cabinas públicas’ (commercial cybercafés), small businesses where computers with reliable Internet connections are rented at low cost, an average of 0.30 U.S. dollars per hour of Internet use.^42^ It is estimated that 70% of the users in Peru access Internet through commercial cybercafés, which makes Peru a country with one of the highest numbers of Internet users in public places.^42,43^

Commercial cybercafés have dramatically increased the Internet access even in Peru’s low income areas.^42^ Some factors, such as the lack of regulation of Web page content, the increasing number of private booths with headphone and webcam availability, and the highly informal atmosphere of these cybercafés make their use for cybersex and real sex more feasible. On the other side, the relative privacy that these cybercafés offer may facilitate the access of participants to online prevention interventions that include sensitive topics such as HIV/STI and homosexuality and also may constitute an alternative venue to physically reach this population.^44^

**C. PRELIMINARY DATA**

In Peru, two previous studies have assessed the role of the Internet as a medium to facilitate sexual encounters as well as accessibility to information about HIV/STI. The first one was a study done among 100 HIV-positive patients who attended an HIV clinic in Lima. Overall, 59% reported using the Internet during the last year, the majority (73%) through a commercial cybercafé. Seeking sex over the Internet was significantly more common among MSM (94% or 15/16) than among Men who have Sex with Women (MSW; 6% or 1/16; p=0.032). All five respondents who reported having had sex with a partner met online were MSM. No women reported having sought or having had sex with an Internet partner during the last year. An important percentage of those who accessed the Internet during the last year (76% or 45/59) use it to find information on HIV and a lower percentage (39% or 23/59) used it to seek information on other STIs. Among those who used the Internet to meet sex partners, 87.5% (14/16) also sought information related to HIV, compared to 72.1% (31/43) not seeking partners (p=0.3). ^45^ This study indicated that was the MSM population the one that should be targeted with risk reduction interventions over the Internet and that the implementation of these interventions in this population may be feasible given the high overlap between seeking sexual partners and also looking for health related information over the Internet.

The second study delivered a Web-based Internet survey from January to March 2006 in a prominent Peruvian Gay Website (GayPeru.com).^3^ In this research, two banner ads linked to identical online surveys were displayed randomly. Both banners invited participants to fill an online survey but only one offered free HIV/syphilis testing and condoms as compensation for participation. This study indicated that it is feasible to collect online data about risk behaviors for HIV and STI in MSM. It also showed that the Internet is an effective tool to reach an important group of high-risk MSM who are not being reached by traditional interventions, have not been tested for HIV, and are interested in receiving web-based prevention interventions. The participants reported favorable attitudes towards online modalities for accessing HIV/STI prevention messages, 93.7% of participants were interested in a Web page, 87.4% in chat rooms, 83.4% in e-mails, 83.1% in e-groups and 60.2% in cell-phone text messages.

Additionally, it was found that free HIV testing can be effectively utilized as an incentive for participation.

1. **METHODS**

**Overview:** We are proposing an effectiveness randomized controlled trial to study the association between an online video-based intervention and proportions of HIV testing in an MSM population in Lima, Peru. The RCT is an effectiveness design as we are not paying participants, in order to reduce response bias and to emulate the conditions that would be necessary for sustained offering of this intervention by the public health sector in Peru. The research plan is divided into (1) the development period, where we will characterize and refine the most effective approaches and the types of messages we will use to reach high-risk MSM online, (2) the intervention, where we will advertise banner ads that will redirect participants into the HIV/STI online risk assessment; eligible participants who agree to participate will be randomized into either a video-based or a text-based intervention and (3) Outcome evaluation, where we will compare the intentions of getting tested and the proportions of HIV testing in each of the trial arms.

**Development Period:** The development period will last 3 months and it will include three main components:

**In-depth face-to-face interviews with key informants**: We will do in-depth interviews with 8 key informants that will include: webmasters of gay websites, gay leaders identified in our previous study and other gay leaders identified by frequent users of Internet sex sites, online discussion moderators and owners of commercial cybercafés where sexual encounters have been reported.

**Focus groups with the target population:** We will conduct two focus groups with each MSM subpopulation: gay and non-gay-identified MSM, and trans (transvestites: i.e., men who cross-dress; transgender and transsexual). The interviews and focus groups will help us to collect information to (1) create a persuasive recruitment message and image for the banner ads, (2) refine the content and format of the website, (3) refine the content of the risk assessment survey and, (4) delineate the script of the video-based intervention.

**Usability tests with regular users of Internet gay websites:** In this period we will do usability tests with 12 regular users of gay websites recruited on the Internet (six novice users and six expert users). Both, the interview and usability tests will be conducted in the Via Libre clinic. During the usability test we will solicit participants’ comments while performing each task (“thinking aloud”) and we will also measure times, wrong pathways and failure to find content

**Intervention:** We will advertise animated banner ads that, if clicked, will redirect the participants to our study website.

**Study website:** The website will be compatible with all popular web browsers. The website will be hosted on a secure server. Our website will include the following five pages: (1) the homepage will contain information about the study and the phone number the participants can call if they need more information, (2) “Who we are” will contain information about the institutions involved and the names of the investigators, (3) “Privacy policy”, will include information about the anonymity of the survey, lack of electronic tracking (no cookies, SSL encryption) and general information about HIV/syphilis testing, and other data security information (4) “Frequently asked questions”, will include questions and answers about the process of participation and the risk and benefits of it, and (5) “Results” will only contain a note to explain that brief, group-level summary results will be available on this page in the future. Each page of the site will contain links to the site’s other pages. There will be one button on the homepage stating “I want to participate”. If the visitor chooses to participate and clicks the button, he will be redirected to a secure portion of the website to complete the risk assessment. If the visitor chooses not to participate he will be presented with a brief questionnaire asking the reasons why he chose not to participate.

**Baseline risk assessment:** The online baseline risk assessment will be designed in LimeSurvey, an open source survey tool. The risk assessment will document demographic characteristics, sexual orientation, sexual and non sexual risk behaviors for STI, presence of STI symptoms, online seeking behavior, history of previous HIV testing and reasons for not taking an HIV-test.

The data collected by the survey will be stored in a relational database hosted on a secure server. Only the survey administrator will have access to the risk assessment data by logging in using an account name and password over a secure connection. Respondents will not be able to access their own or anyone else’s results. In the questionnaires, participants will not be asked for any personally identifiable information. However, they will be asked to provide their email address. This email address will be stored in the database and will allow us to identify the experimental condition to which participants who come to our site to be tested have been randomized..

**Eligibility Criteria:** In order to be randomized to one of the interventions the participants have to fulfill all of the following eligibility criteria: (1) be 18 years of age or older, (2) be a man and report having had sex with men, (3) be a resident of Lima, Peru, (4) answer the survey from Lima, Peru (5) not have been tested for HIV during the last year, (5) have an email address that when typed twice matched and, (6) do not report being HIV positive. All of these criteria will be evaluated during the risk assessment

**Randomization:** After the participant finishes the baseline risk assessment and enters his email address, our system will immediately assess the validity of this email and the fulfillment of all the eligibility criteria. Only if both requirements are met will the computer randomly assign the participant to one of the two arms of the intervention (Figure 1). The randomization will be automatically done by an algorithm that evaluates each case and uses a random number generator to make an independent assignment. The resulting assignments should be roughly fifty percent to the intervention group and fifty percent to the control group.

**Types of interventions**

**Video-based intervention:** This intervention will provide a message to motivate participants to get tested for HIV. This message will be customized for three audiences based on self-identification: gay, non-gay men and trans. The videos will be framed within the health-belief model, which focuses on the attitudes and beliefs of individuals, and within the stages of change theory, which shows that people tend to progress through different stages on their way to successful behavioral change [46,47]. The videos will incorporate ways to overcome different reasons why MSM don’t get tested for HIV, the script of the videos will be refined during focus groups with the target population.

**Text-based intervention:** This intervention will provide a standard public health text message with an invitation to a free HIV/syphilis testing, the message will be similar in content to Web pages currently available at Latin American websites.

**Phase III: Evaluation of Outcome measures:**

In this phase we will compare the intentions to get tested and the proportion of participants tested for HIV from each of the study arms. The email addresses that participants self-reported on the online survey will be matched with those self-reported at the study site to allow us the identification of the experimental condition to which participants were randomized. For the analysis, the results of the surveys will be exported from the survey database into SPSS 13.0 software. We will use Mantel Hanzel to calculate the Relative Risk of intentions to get tested for HIV and Cox proportional hazards regression to calculate the Hazard Ratios of getting tested at our clinic among the study arms of the intervention. We will follow the intention to treat principle.

Consent form and Screening questionnaire

Exclusion

Do not meet inclusion criteria

Do not want to participate

Randomization

Allocated and received video-based intervention

Allocated and received text-based intervention

**Figure 1: Randomization Process**

**Sample size determination**

Our hypothesis is that a video-based intervention compared to a text-based intervention will increase the proportion of HIV testing by 12%. Assuming a proportion of HIV testing of 3.0% in our control intervention and 15.0% in our active intervention, our estimated sample size is 87 participants for each arm of the intervention. This will provide a good power (0.80, at alpha 0.05) to see an absolute difference of 12% in testing proportions between the two arms.

## Limitations:

## Some limitations of our study are: (1) limited generalizability of our results due to the fact that we will recruit a convenience sample (2) selection bias in terms of educational background and age (3) inability to collect data about participants who attended other clinics to receive testing (4) possible self-misrepresentation of some participants leading to misclassification (e.g., a female answering as a male) and (5) inability to measure compliance with the intervention.

## E. Project timeline

| **Project Activities** | **2007** | | | **2008** | | |
| --- | --- | --- | --- | --- | --- | --- |
|  | **April-June** | **July-Sep** | **Oct-Dec** | **Jan-Mar** | **April-Jun** | **Jul-Sep** |
| Administrative preparation (finalize IRB approvals) | *** |  |  |  |  |  |
| Selection Gay websites, focus groups and pilot tests |  | *** |  |  |  |  |
| Recruitment of participants |  |  | *** | *** |  |  |
| Follow-up of participants |  |  | *** | *** | *** |  |
| Data analysis & manuscript preparation |  |  |  |  |  | *** |

## F. Protection of Human Subjects

Our grant proposal will be sent for review to the Institutional Review Board of the University of Washington in Seattle and the NGO Vía Libre in Lima, Peru.

Human Subjects Involvement and Characteristics: We will recruit through Internet MSM 18 years and older who voluntarily are willing to participate in our study and meet the following eligibility criteria: (1) be 18 years of age or older, (2) be a man and report having had sex with men, (3) be a resident of Lima, Peru, (4) answer the survey from Lima, Peru (5) not have been tested for HIV during the last year, (5) have an email address that when typed twice matched and, (6) do not report being HIV positive.

Collection of information: On the Internet, data will be collected via a secure website. The data from the survey will be kept confidential. Only authorized personnel will access the data by logging in using an account name and password. Data that have been exported into statistical software will be stored on an access-controlled computer in a locked room at Via Libre.

Respondents will not be able to access their own or anyone else’s survey. At the homepage of our study, we will tell the participants that they may skip any question they choose not to answer and that the testing for HIV is voluntary and they may decide not to come.

During the survey, respondents will be asked to enter their email address for storage in the database. This email will be later used in the clinic to identify to which arm of the intervention a participant belongs. This will be possible only if the participant discloses his email address through a written informed consent in our site.

Potential Risks: Potential risks of answering the questionnaire include feelings of discomfort or other psychological distress arising from the personal nature of some questions, and the internal conflicts that some men may experience between their values, attitudes, beliefs and behaviors. Potential risks of being tested for HIV include stress, anxiety and depression. If the HIV results are negative it can increase the percentage of unsafe sex which would increase the risk of becoming infected with HIV or other STI. If the tests are positive and get into the wrong hands, the participants may lose their job.

The needle used to draw blood for the tests may cause pain and participants may get a bruise after the procedure.

Recruitment and Informed Consent: Banner ads will run on prominent Peruvian gay websites. The message from the banner ads will be refined during the focus groups. The participants who complete the self-assessment and are randomized on either of the 2 arms of the intervention will have the option of voluntarily go to Vía Libre to get the free tests. Upon arriving at Vía Libre, the participant will be referred to the study coordinator who will explain the risk and benefits of the HIV tests and how the information he provided on the baseline risk assessment will be linked to the arm of the intervention he belongs. After the participant signs the Informed Consent accepting to be tested for HIV and syphilis and accepting to disclose his email, the counselor will perform the pre-test for HIV and collect the email of the participant. Then the participant will be provided with 20 condoms and directed to the laboratory. There, the lab technician will perform a rapid HIV test. If a participant has a positive rapid test for HIV, we will do an ELISA test and then a confirmatory test using Western Blot. We will not ask complete names, addresses, phone numbers, ID numbers or any other form of identification. The results of the HIV and syphilis tests will be stored in a locked cabinet.

Protection against risk: In order to minimize risks of harm and to protect subjects’ rights and welfare, subjects will be provided with our business hours, the email address and telephone number of Vía Libre where they can call if they become distressed and want to talk.

In our study the possibility of discovering suicidal intentions does exist. Should this occur, staff are prepared to address the situation. At Vía Libre there are physicians that can respond in an emergency situation and psychologists that can assess for suicide risk and, if warranted, engage the caller in a no-harm contract and locate appropriate local referral resources. We will follow-up as indicated until the situation is stabilized.

Potential benefits of the proposed research to the subjects and others: An important benefit of the study participants is that they will have the option of getting tested for HIV, syphilis, and receive counseling as well as condoms at no cost, if they are worried about AIDS or any other STI, they might feel better if they receive a negative result. Additionally, knowing a result can also help to diagnose a medical problem or guide health care.

Importance of the Knowledge to Be Gained: The benefits of this research for society could be substantial; the Internet holds great potential not only as a survey research tool, but as a venue for delivering easily accessible, high quality, intervention services. Internet use in Peru is widely available even to people with limited resources. If our work is successful, the approach can be adapted to other populations affected by HIV especially those from developing countries.

**III. REFERENCES**

1. Tabet S SJ, Lama J, Goicochea P, Campos P, Rouillon M, Cairo JL, Ueda L, Watts D, Celum C, Holmes KK. HIV, syphilis and heterosexual bridging among Peruvian men who have sex with men. AIDS 2002;16(9):1271-7.

2. Kusunoki L, Guanira J, Navarro C, Velasquez C. Report of Monitoring the declaration of commitment on HIV/AIDS. December 2005 [document on the Internet]. 2006 [cited 2006 May 20]; Available from: <http://www.planvihperu.org/index.php?option=com_content&task=view&id=28&Itemid=49>

3. Blas M, Alva I, Cabello R, Garcia P, Redmon M, Kurth A, Kimball AM, Ryan R. Response to an Internet Survey of Men Who Have Sex with Men in Peru In Mednet 2006: 11th World Congress on Internet in Medicine. Toronto Canada, October 13-20 2006 Available from: <http://www.mednetcongress.com/ocs/viewabstract.php?id=330>

4. UNAIDS. AIDS epidemic update [document on the Internet]. December 2005. 2006 [cited 2006 May 20]; Available from: <http://www.unaids.org/epi/2005/doc/EPIupdate2005_pdf_en/epi-update2005_en.pdf>

5. Ministry of Health. OGE. Bulletin of Epidemiology [document on the Internet]. March 2006 [cited 2006 May 20]; Available from: [http://www.oge.sld.pe/vih/Boletin%202006/marzo.pdf](http://www.oge.sld.pe/vih/Boletin 2006/marzo.pdf)

6.Lama JR, Lucchetti A, Suarez L, Laguna-Torres VA, Guanira JV, Pun M, Montano SM, Celum CL, Carr JK, Sanchez J, Bautista CT, Sanchez JL; Peruvian HIV Sentinel Surveillance Working Group. Association of herpes simplex virus type 2 infection and syphilis with human immunodeficiency virus infection among men who have sex with men in Peru. J Infect Dis. 2006 Nov 15;194(10):1459-66.

7. Eng T, Gustafson DH, Henderson J, Jimison H, Patrick K. Introduction to evaluation of interactive health communication applications. American Journal of Preventive Medicine 1999;16(10-15).

8. Rietmeijer CA, Bull SS, McFarlane M, Patnaik JL, Douglas JM, Jr. Risks and benefits of the Internet for populations at risk for sexually transmitted infections (STIs): results of an STI clinic survey. Sex Transm Dis 2003;30(1):15-9.

9. Bull SS, McFarlane M, King D. Barriers to STD/HIV prevention on the Internet. Health Educ Res 2001;16(6):661-70.

10. Benotsch EG, Kalichman S, Cage M. Men who have met sex partners via the Internet: prevalence, predictors, and implications for HIV prevention. Arch Sex Behav 2002;31(2):177-83

11. McFarlane M, Bull SS, Rietmeijer CA. The Internet as a newly emerging risk environment for sexually transmitted diseases. JAMA 2000;284(4):443-6.

12. Elford J, Bolding G, Sherr L. Seeking sex on the Internet and sexual risk behaviour among gay men using London gyms. AIDS 2001;15(11):1409-15.

13. Cohn Susan, Rebecca C. Human Immunodeficiency Virus Infection in Women. In: Mandell GL BJ, Dolin R, editor. Mandell, Douglas, and Bennett's principles and practice of infectious diseases. New York: Elsevier/Churchill Livingstone; 2005. p. 1616 - 38.

14. Centers for Disease Control and Prevention (CDC). Internet use and early syphilis infection among men who have sex with men-San Francisco, California, 1999-2003.MMWR Morb Mortal Wkly Rep. 2003 Dec 19;52(50):1229-32.

15. Klausner JD, Wolf W, Fischer-Ponce L, Zolt I, Katz MH. Tracing a syphilis outbreak through cyberspace. JAMA 2000;284(4):447-9.

16. Tashima KT, Alt EN, Harwell JI, Fiebich-Perez DK, Flanigan TP. Internet sex-seeking leads to acute HIV infection: a report of two cases. Int J STD AIDS 2003;14(4):285-6.

17. Centers for Disease Control and Prevention (CDC). Using the Internet for partner notification of sexually transmitted diseases. Los Angeles County, California, 2003. MMWR Morb Mortal Wkly Rep. 2004 Feb 20;53(6):129-31.

18. Ross MW. The Internet as a medium for HIV prevention and counseling. Focus. 2002 Apr;17(5):4-6.

19. Levine DK, Scott KC, Klausner JD. Online syphilis testing--confidential and convenient.Sex Transm Dis. 2005 Feb;32(2):139-41.

20. Bull SS, Lloyd L, Rietmeijer C, McFarlane M. Recruitment and retention of an online sample for an HIV prevention intervention targeting men who have sex with men: the Smart Sex Quest Project. AIDS Care. 2004 Nov;16(8):931-43.

21. Bowen AM, Horvath K, Williams ML. A randomized control trial of Internet-delivered HIV prevention targeting rural MSM. Health Educ Res. 2006 Jul 18.

22. Pequegnat W, Rosser BR, Bowen AM, Bull SS, Diclemente RJ, Bockting WO, Elford J, Fishbein M, Gurak L, Horvath K, Konstan J, Noar SM, Ross MW, Sherr L, Spiegel D, Zimmerman R. Conducting Internet-Based HIV/STD Prevention Survey Research: Considerations in Design and Evaluation. AIDS Behav. 2006 Oct 20

23. Ross MW, Tikkanen R, Mansson SA. Differences between Internet samples and conventional samples of men who have sex with men: implications for research and HIV interventions. Soc Sci Med 2000;51(5):749-58.

24. Flatley-Brennan P. Computer network home care demonstration: a randomized trial in persons living with AIDS. Comput Biol Med. 1998 Sep;28(5):489-508.

25. Safren SA, Hendriksen ES, Desousa N, Boswell SL, Mayer KH. Use of an on-line pager system to increase adherence to antiretroviral medications. AIDS Care. 2003 Dec;15(6):787-93.

26. Bull SS, Lloyd L, Rietmeijer C, McFarlane M. Recruitment and retention of an online sample for an HIV prevention intervention targeting men who have sex with men: the Smart Sex Quest Project. AIDS Care. 2004 Nov;16(8):931-43.

27. Bowen AM, Horvath K, Williams ML. A randomized control trial of Internet-delivered HIV prevention targeting rural MSM. Health Educ Res. 2006 Jul 18.

28. Bezerra A, Souza C, Abbate M, Abreu L. Integrating prevention through virtual communication. In the XV International AIDS Conference. Bangkok Thailand, July 11-16 2004. Available from: <http://www.iasociety.org/abstract/show.asp?abstract_id=2176023>

29. Ramos M, Germany C, D’Elia P, Harzheim E, Vissoki J, Valdez I, Viana J. Dynamic, free of charge, Internet platform as a tool to facilitate access to STI/HIV care in Porto Alegre, Brazil. In the XV International AIDS Conference. Bangkok Thailand, July 11-16 2004. Available from: <http://www.iasociety.org/abstract/show.asp?abstract_id=2167641>

30. Chaika N. Internet as information source for Russian HIV/AIDS specialist and NGO. In the XIV International AIDS Conference. Barcelona, Spain July 7-12 2002. Available from: <http://www.iasociety.org/abstract/show.asp?abstract_id=2292>

31. Huynh H. Sharing knowledge in HIV-prevention and care thanks to information technology to protect our Vietnamese communities. In the XIV International AIDS Conference. Barcelona, Spain July 7-12 2002. Available from: <http://www.iasociety.org/abstract/show.asp?abstract_id=5260>

32. Luna M, Quintero P, Montaño M, Rodríguez A, Garcia A, Zamudio I, Salazar M,  Salazar S, Quintero K, Pascual, Valdes M. Coffee health and VIHDA, first site interactive coffee tematic on AIDS/HIV and others STDs. In the XV International AIDS Conference. Bangkok Thailand, July 11-16 2004. Available from: <http://www.iasociety.org/abstract/show.asp?abstract_id=2168179>

33. Abaleña. Electronic connectivity: An IEC intervention for HIV/AIDS prevention among the youth. In the XV International AIDS Conference. Bangkok Thailand, July 11-16 2004. Available from: <http://www.iasociety.org/abstract/show.asp?abstract_id=2172292>

34.Joel O, Okoro O, Shobande J, Klindera K, Franka A, Penn A, Mbanjwa P, Oteng F. The web: an effective tool for promoting HIV/AIDS among young people in Nigeria. In the XV International AIDS Conference. Bangkok Thailand, July 11-16 2004. Available from:
<http://www.iasociety.org/abstract/show.asp?abstract_id=2174471>

35. Sescon J, Rada P, Papua G. Youth zone Internet chat counseling: Learnings gained in integrating Internet. In the XIV International AIDS Conference. Barcelona, Spain July 7-12 2002. Available from: <http://www.iasociety.org/abstract/show.asp?abstract_id=7800>

36. Monareng G, Ramalaga S, Maluleke S, Klindera K, Oteng F. Cyber Café development as a HIV/AIDS prevention strategy. In the XV International AIDS Conference. Bangkok Thailand, July 11-16 2004. Available from: <http://www.iasociety.org/abstract/show.asp?abstract_id=2175425>

37. Mayorga R, Amaya K, López Sologaistoa J. OASIS electronic information center: a learning tool for HIV/AIDS prevention. . In the XIV International AIDS Conference. Barcelona, Spain July 7-12 2002. Available from:

<http://www.iasociety.org/abstract/show.asp?abstract_id=7586>

38. Carr R, McKnight I, Lewis S. Breaking history: Building a network of gay and other men who have sex with men (MSM) across the small islands of the Caribbean. In the XV International AIDS Conference. Bangkok Thailand, July 11-16 2004. Available from: <http://www.iasociety.org/abstract/show.asp?abstract_id=2175943>

39. Castro S, Bauer P, Biondi E, Santos J, Veiga L, Santos A, Silva L, Souza A. An official Web page for broad information about HIV/AIDS, STDs, hepatitis, tuberculosis and drug use in the penitentiary system of Rio de Janeiro (Brazil). In the XIV International AIDS Conference. Barcelona, Spain July 7-12 2002. Available from: <http://www.iasociety.org/abstract/show.asp?abstract_id=4016>

40. Wang Q, Lin G, Ross M. Sexual risk behaviors among men who have sex with men in China: an Internet survey. In the XV International AIDS Conference. Bangkok Thailand, July 11-16 2004. Available from: <http://www.iasociety.org/abstract/show.asp?abstract_id=2172290>

41. Internet World Stat Internet Usage Statistics for the Americas. North America Internet Users and Population Stats. 2007. Available from: [http://www.internetworldstats.com/stats2.htm#south](http://www.internetworldstats.com/stats2.htm#south)

42. Fernandez-Maldonado, A., Las cabinas públicas de Internet en el Perú. Perfil de los usuarios y los usos. 2000.

43. Villanueva, E., Internet availability and politics in Peru: a preliminary report on an apparent paradox. 2004, Departamento de Comunicaciones Pontificia Universidad Católica del Perú.

44. Curioso WH, Blas M, Nodell B, Alva I, Kurth A. Opportunities for providing Web-based interventions for sexually transmitted infection prevention in Peru’s Internet cafes. PLoS Medicine. In press.

45. Alva IE, Blas MM, Garcia PJ, Cabello R, Kimball AM, Holmes KK. Risks and benefits of Internet use among people living with HIV/AIDS in Lima, Peru. Rev Med Exp Sal Pub 2007;(3).

46. DiClemente, C.C., Prochaska, J.O., Fairhurst, S.K., Velicer, W.F., Velasquez, M.M. et al. The process of smoking cessation: an analysis of precontemplation, contemplation, and preparation stages of change. J Consult Clin Psychol 59, 295-304(1991).

47. Prochaska, J.O., Velicer, W.F., Rossi, J.S., Goldstein, M.G., Marcus, B.H., et al. Stages of change and decisional balance for 12 problem behaviors. Health Psychol 13, 39-46(1994).
