# Supplementary material for: Effect of an Online Video-Based Intervention to Increase HIV Testing in Men Who Have Sex with Men in Peru
Source: PLoS One. 2010 May 3;5(5):e10448. doi: 10.1371/journal.pone.0010448 (PMC2862715; doi:10.1371/journal.pone.0010448)
Supplement: Spanish S1 — Spanish translation of the article (0.25 MB DOC) [file pone.0010448.s003.doc]

**Uso de videos en Internet para fomentar el despistaje de infección por VIH en hombres que tienen sexo con hombres: Un estudio aleatorizado**

**Magaly M. Blas, Isaac E. Alva, César P. Cárcamo, Robinson Cabello, Steven M. Goodreau, Ann M. Kimball y Ann E. Kurth**

Magaly M. Blas es de la Unidad de Epidemiología, VIH y ETS, Facultad de

Salud Pública y Administración, Universidad Peruana Cayetano Heredia, Lima, Perú, de la ONG Vía Libre, Lima, Perú y del Departamento de Epidemiología, Escuela de Salud Pública y Medicina Comunitaria de la Universidad de Washington, Seattle, Washington, Estados Unidos de América

Isaac E. Alva es de la Unidad de Epidemiología, VIH y ETS, Facultad de

Salud Pública y Administración, Universidad Peruana Cayetano Heredia, Lima, Perú,

y de la ONG Vía Libre, Lima, Perú

César P. Cárcamo es de la Unidad de Epidemiología, VIH y ETS, Facultad de

Salud Pública y Administración, Universidad Peruana Cayetano Heredia, Lima, Perú

Robinson Cabello, es de la ONG Vía Libre, Lima, Perú

Steven M. Goodreau es del Departamento de Antropología de la Universidad de Washington, Seattle, Washington, Estados Unidos de América

Ann M. Kimball es del Departamento de Epidemiología, Escuela de Salud Pública y Medicina Comunitaria de la Universidad de Washington, Seattle, Washington, Estados Unidos de América

Ann E. Kurth es de los Departamentos de Salud Global, y de Epidemiología, Escuela de Salud Pública y Medicina Comunitaria, y de la Escuela de Enfermería de la Universidad de Washington, Seattle, Washington, Estados Unidos de América

**Resumen**

**Antecedentes:** A pesar de que un alto número de hombres que tienen sexo con hombres (HSH) en el Perú desconocen su status de VIH, muchos de ellos son usuarios frecuentes de Internet, y pueden ser motivados a través de este medio a hacerse la prueba de VIH.

**Métodos:** Se realizó un ensayo controlado aleatorizado a través de Internet para comparar el efecto de un video versus un texto estándar de salud pública, ambas intervenciones motivaban a los HSH a hacerse la prueba de VIH y fueron difundidos a través de sitios web gay. Se preparó un video dirigido a HSH autoidentificados como gay, y otro dirigido a HSH no autoidentificados como gay. Los resultados evaluados fueron: (i) intención de hacerse la prueba de VIH y (ii) visita a nuestra clínica para hacerse la prueba de VIH.

**Resultados:** En el grupo de HSH no identificados como gay, 97 hombres recibieron la intervención basada en video y 90 la intervención basada en texto. Quienes recibieron el video reportaron con más frecuencia su intención de hacerse la prueba de VIH en los próximos 30 días (62.5% vs. 15.4%, RR: 2.77, IC 95%: 1.42-5.39). Once participantes asignados al video y ninguno de los participantes asignados al texto acudieron a nuestra clínica a hacerse la prueba de VIH (p = 0.001). En el grupo identificado como gay, 142 hombres fueron asignados a la intervención basada en video y 130 a la intervención basada en texto. Aquellos asignados al video reportaron con mayor frecuencia tener intenciones de hacerse la prueba de VIH dentro de los próximos 30 días, aunque no de forma significativa (50% vs. 21.6%, RR: 1.54, IC 95%: 0.74-3.20). Al final del seguimiento, 8 participantes que vieron el video y 10 que leyeron el texto visitaron nuestra clínica para hacerse la prueba de VIH (Razón de peligro: 1.07, IC 95%: 0.40-2.85).

**Conclusión:** Este estudio proporciona evidencia de la eficacia de una intervención basada en un video difundido a través de Internet, en el incremento de las pruebas de VIH entre HSH no identificados como gay en el Perú. Esta intervención podría ser adoptada por instituciones con sitios web orientados a motivar a que poblaciones similares de HSH se hagan la prueba de VIH.

Registro de ensayos:

Effect of an Online Video-Based Intervention to Increase HIV Testing in Men Who Have Sex With Men in Peru; número de registro NCT00751192;

<http://clinicaltrials.gov/ct2/show/NCT00751192?term=NCT00751192&rank=1>

**Introducción**

En el año 2007, 58,000 personas perdieron la vida debido al SIDA y aproximadamente 100,000 se infectaron con VIH en América Latina. Un factor de riesgo importante en las epidemias de varios países de esta región es el sexo sin protección entre hombres que tienen sexo con hombres (HSH). Los países con epidemias de este grupo son El Salvador, Guatemala, Honduras, México, Nicaragua y Panamá en América Central y Bolivia, Chile, Ecuador y el Perú en América del Sur [1]. Como resultado, el impacto potencial de reducir el riesgo en esta población es significativo.

En el Perú, los programas actuales dirigidos a los HSH se basan en intervenciones a través de educadores de pares que acuden a calles, saunas y  otros lugares frecuentados por la población HSH. Desafortunadamente, este enfoque sólo alcanza entre el 18% y 45% de la población HSH, y deja de lado poblaciones menos propensas a ser identificadas como gay, como HSH ocultos, HSH jóvenes y bisexuales [2 - 4].

Con el fin de reducir la transmisión del VIH entre los HSH, se debe promover la identificación y tratamiento temprano del VIH y otras infecciones de transmisión sexual (ITS). Un estudio realizado en el 2006 en Lima, demostró que es factible llegar a los HSH de alto riesgo a través de Internet [5-6]. La investigación demostró que la tasa de participación en una encuesta dirigida a HSH a través de Internet se incrementó cuando se ofreció pruebas de VIH/sífilis gratuitas como única compensación por la participación. Esta encuesta también atrajo HSH de alto riesgo que no se habían hecho pruebas de VIH y que estaban interesados en una amplia variedad de intervenciones de prevención basadas en Web [6,7].

En general, las intervenciones basadas en Internet han demostrado ser un método eficaz para proporcionar información sobre la salud a un gran número de personas que reportan comportamientos sexuales de alto riesgo y que de otro modo no buscarían atención y no serían abordados por intervenciones de promoción de la salud [8]. Varias estrategias a través de Internet han tenido cierto éxito, por ejemplo: la creación de sitios educativos, intervenciones de reducción de riesgo, notificación de pareja, sesiones de consejería y facilitación de pruebas para ITS. [9-12]. Además, recientes intervenciones basadas en video (con y sin el uso de Internet), han demostrado ser eficaces en la disminución de conductas de riesgo para el VIH y en la reducción de la adquisición de ITS [13-14]. Estos factores, sumados a la importancia de la detección temprana del VIH, y la reciente introducción del tratamiento antirretroviral de gran  actividad (TARGA) gratuito en el Perú, hacen imperativo el desarrollo y evaluación de intervenciones basadas en videos difundidos a través de Internet para incrementar el número de HSH de alto riesgo que se hacen la prueba de VIH.

Dada la necesidad de priorizar recursos de salud pública en países en desarrollo, hemos propuesto un ensayo aleatorizado controlado para estudiar la asociación entre las intervenciones basadas en video a través de Internet y la frecuencia de pruebas de VIH en HSH identificados o no como gay.

Métodos

El protocolo de este estudio y la declaración CONSORT están disponibles como información adicional; ver Protocol S1, Checklist S1. También está disponible una versión en inglés del artículo.

De octubre del 2007 a abril del 2008, se realizó un ensayo aleatorizado (EA) para comparar la eficacia de dos intervenciones difundidas a través de sitios web gay peruanos: un video versus un texto estándar desarrollados para motivar a los HSH a hacerse la prueba de VIH.

Cinco de los sitios web gay utilizados eran comerciales (gayperu.com, peruesgay.com, diariodelimagay.com, deambiente.com, chicoslima.com) y dos eran sitios web de abogacía (mhol.org.pe y runa.org.pe).

Este estudio se realizó de acuerdo con los principios expresados en la Declaración de Helsinki. El protocolo del estudio fue aprobado por el comité de ética de la Universidad de Washington en Seattle y de la organización no gubernamental Vía Libre en Lima, Perú. Todos los participantes llenaron un consentimiento informado electrónico para el cuestionario en Internet y un consentimiento informado por escrito para la toma de muestras.

Investigación formativa

A fin de desarrollar la intervención, se realizaron ocho entrevistas a profundidad  y dos grupos focales con cada subpoblación HSH: aquellos identificados como gay, aquellos no identificados como gay y trans (travestis, transgéneros y transexuales).

Durante este período se hizo evidente que un video describiendo una historia y un personaje con el que los participantes se puedan identificar era más atractivo que uno con personajes animados. Además, los grupos focales ayudaron a identificar las razones principales por las que los HSH no se hacen la prueba de VIH, así como los personajes y las historias que deberían presentarse en los videos. También identificamos la necesidad de agregar subtítulos a los videos para facilitar la privacidad en las cabinas públicas y para permitir el acceso a los videos en equipos sin audio disponible.

Además, en este período se hizo evidente la necesidad de personalizar el video de acuerdo a la auto-identificación: gay, no-gay y trans. En un diseño de abordaje centrado en el usuario [15], involucramos a miembros de la comunidad, activistas e investigadores en el proceso de elaboración y edición de post-producción del video. Finalmente, realizamos una prueba piloto con la población objetivo.

Avisos publicitarios

Publicamos avisos animados que redirigían a los participantes a la página web de nuestro estudio. Los avisos incluyeron el siguiente mensaje: 'SOMOS' (que era el nombre del proyecto y el acrónimo de  Servicios Óptimos para Mejorar la Oportunidad en Salud), "¿sexo?", "Responde nuestra encuesta anónima” y “haz clic aquí”. En el anuncio, no incluimos una referencia a "la prueba de VIH" dado que es probable que esto hubiese atraído sólo a los participantes interesados en realizarse pruebas y por lo tanto hubiese reducido el poder para detectar una diferencia entre los dos brazos de la intervención.

Página Web del estudio:

Nuestra página Web incluyó información sobre los riesgos y beneficios de la participación, información sobre nuestra política de privacidad, preguntas frecuentes, un número de teléfono para que los participantes llamen si necesitan más información y un botón con un enlace a la encuesta en línea para aquellos que deseaban participar.

La encuesta fue diseñada usando LimeSurvey [16], y documentó las características demográficas, orientación sexual, comportamientos de riesgo sexuales y no sexuales con respecto a las ITS, presencia de síntomas de ITS, búsqueda de sexo a través de Internet, historia de pruebas de VIH previas, razones para no hacerse la prueba de VIH, y el estadio de cambio/disposición de los participantes para hacerse la prueba de VIH. En los cuestionarios, no se pidió ninguna  información que pueda identificar a los participantes, pero se les pidió que introduzcan su dirección de correo electrónico dos veces (se les sugirió dar una dirección anónima), que fue utilizada para conectar a cada participante que asistió a la clínica con su datos de cuestionario y el brazo de la intervención al que fue asignado.

Criterios de inclusión

Para ser asignados al azar a una de las intervenciones, los participantes tenían que cumplir todos los siguientes criterios de elegibilidad: (1) tener 18 años de edad o más, (2) ser hombre y reportar haber tenido relaciones sexuales con otro hombre, (3) ser residente de Lima, (4) responder a la encuesta en Lima, (5) no haberse hecho pruebas de VIH durante el último año, (6) haber ingresado dos veces una misma dirección de correo electrónico, y (7) reportar no ser VIH positivo.

Con el fin de evitar que participantes no elegibles sean aleatorizados, no hicimos evidente los criterios de inclusión. Además, permitimos ver el contenido de la encuesta a personas no interesadas en participar en ella. Por último, a fin de reducir el sesgo de participación, no ofrecimos incentivos económicos para el llenado de la encuesta. Después de terminar la evaluación basal de riesgo e ingresar su dirección de correo electrónico dos veces, nuestro sistema evaluó el cumplimiento de todos los criterios de elegibilidad del participante. Al participante se le invitó a participar en la intervención sólo si  cumplía todos estos criterios.

Tipos de intervenciones

Una vez que el participante estuvo de acuerdo con participar en la intervención y cumplió todos los criterios de elegibilidad, se le presentó al azar un video o un texto. Un algoritmo en una computadora evaluó cada caso y usó un generador de números aleatorios para seleccionar de manera independiente la intervención para cada participante.

Los videos tenían una duración de cinco minutos y fueron personalizados para población HSH en tres categorías basadas en su auto-identificación: no identificados como gay (Video S1), identificados como gay (Video S2), e identificados como trans (Video S3) [17-19]. En este artículo presentamos sólo los resultados de los dos primeros grupos, porque sólo 21 personas se identificaron con la tercera categoría, muestra que no proporciona poder suficiente para encontrar diferencias entre las intervenciones. El video dirigido a los HSH identificados como gay se presentó si el participante se identificó como "gay" o "caleta" (gays que permanecen total o parcialmente ocultos). El video dirigido a los HSH no identificados como gay fue presentado si la auto-identificación del participante fue heterosexual, bisexual, o "flete" (varones que ofrecen servicios sexuales a otros hombres a cambio de dinero). Dado que no hubo ninguna intervención para incrementar las pruebas de VIH a través de Internet en el Perú, los participantes asignados al texto recibieron una intervención adaptada de una intervención en curso para incrementar las pruebas de VIH en México [20].

Los videos se enmarcaron dentro del modelo de creencias de salud, que se centra en las actitudes y creencias de los individuos, y dentro de la teoría de estadios de cambio, que muestra que las personas tienden a avanzar por diferentes etapas en su camino hacia el cambio de comportamiento exitoso [21,22].

Los videos incorporaron formas de superar las siguientes razones por las que los HSH no se hacen la prueba de VIH: (1) miedo a las consecuencias de un resultado positivo, (2) sensación de nunca haber estado en riesgo de infección, (3) temor a la discriminación, (4) temor a la falta de confidencialidad del personal de salud, (5) temor al rechazo de familiares / amigos / pareja, (6) desconocimiento sobre lugares dónde hacerse la prueba, y (7) falta de solvencia económica para pagar la prueba de VIH, o (8) el tratamiento para VIH.

Las historias mostradas en los videos muestran al personaje principal a través de los diferentes estadios de cambio de pre-contemplación, contemplación, preparación para hacerse la prueba y acción (hacerse la prueba). Ambos, el video y el texto motivaron a los participantes a visitar nuestra clínica para hacerse la prueba de VIH de forma gratuita.

Evaluación de los resultados:

La teoría de estadios de cambio fue usada para clasificar el estadio de los participantes con respecto al comportamiento positivo de hacerse la prueba de VIH [22]. Si los participantes reportaron que no tenían previsto hacerse la prueba de VIH en los próximos 6 meses fueron clasificados en el estadio de pre-contemplación. Si tenían la intención de hacerse la prueba de VIH en los próximos 6 meses estuvieron en el estadio de contemplación. Si ellos indicaron que tenían previsto hacerse la prueba de VIH en los próximos 30 días, fueron clasificados en el estadio de preparación. Los participantes que se hicieron la prueba en el último año podrían estar en el estadio de acción o mantenimiento, y fueron excluidos del estudio.

Los resultados evaluados fueron los cambios de comportamiento de un estadio previo al  siguiente: del estadio de pre-contemplación al de contemplación, del estadio de contemplación al de preparación y de cualquier estadio al de acción.

Análisis Estadístico

Para calcular el tamaño de la muestra asumimos que una intervención basada en video en comparación con una intervención basada en texto aumentaría la proporción de pruebas de VIH en un 12%. Asumiendo una proporción de pruebas de VIH del 3.0% en la intervención basada en texto y 15.0% en la intervención basada en video, el tamaño de muestra requerido es de 87 participantes por cada brazo de la intervención, para un poder de 80% y una  significancia de 95%. Los investigadores permanecieron ciegos a la asignación de tratamiento hasta que cada participante culminó los procedimientos del estudio.

Para evaluar la efectividad de las intervenciones, comparamos la proporción de participantes que se hicieron la prueba de VIH de cada uno de los brazos del estudio utilizando un análisis de intención de tratar. Las direcciones de correo electrónico que los participantes reportaron en la encuesta en línea fueron comparadas con las reportadas por ellos cuando acudieron a nuestra clínica a hacerse la prueba de VIH. Esto nos permitió identificar el grupo experimental al que los participantes fueron asignados aleatoriamente. Para probar la hipótesis de que la intervención basada en video fue más eficaz que la intervención basada en texto utilizamos riesgos relativos ajustados por el método de Mantel Haenszel. Medimos las diferencias en la proporción de participantes que (i) planearon hacerse la prueba de VIH en los próximos 6 meses, (ii) que planearon hacerse la prueba de VIH dentro de los próximos 30 días, (iii) que sacaron una cita por Internet para acudir a nuestra clínica y (iv) que acudieron a nuestra clínica.

Para probar la hipótesis de que la intervención basada en video comparada con la intervención basada en texto aumentó la probabilidad de hacerse la prueba de VIH en nuestra clínica utilizamos análisis de supervivencia, calculando las razones de peligro ajustadas por regresión de Cox. El análisis se realizó en Stata v8.0.

Dado que la intervención y la asignación al azar fueron diferentes para las poblaciones identificadas y no identificadas como gay, el análisis se llevó a cabo de forma independiente para cada uno de los dos ensayos.

Resultados

Recibimos 1,481 encuestas. De éstas, 808 fueron de participantes que se identificaron como gay, 588 de participantes que no se identificaron como gay, 21 de participantes identificados como  trans y el resto fue de mujeres y hombres heterosexuales (Figura 1). Reportamos  sólo los resultados del grupo de HSH identificados como gay y no identificados como gay.

Características de los Participantes

En el grupo de HSH no identificados como gay, 97 hombres fueron asignados aleatoriamente a la intervención basada en video y 90 a la intervención basada en texto. En el grupo identificado como gay, 142 hombres fueron asignados a la intervención basada en video y 130 a la intervención basada en texto (Figura 1). En el grupo de HSH no identificados como gay, no hubo diferencias significativas en las características demográficas de los participantes de la intervención basada en video y de la intervención basada en texto. Sin embargo, en el grupo de HSH identificados como gay, los participantes de la intervención basada en video tuvieron más edad que los participantes de la intervención basada en texto (p = 0.04, Tabla 1).

En  el grupo de HSH no identificados como gay, tampoco hubo diferencias en la orientación sexual, la auto-identificación, el  rol sexual y la información sobre VIH recibida durante el último año de los participantes de la intervención basada en video y de la intervención basada en texto. Sin embargo, en el grupo de HSH identificados como gay, los participantes de la intervención basada en video reportaron un mayor porcentaje de bisexualidad que los participantes de la intervención basada en texto (p = 0.05, Tabla 1). En las dos ramas de intervención del grupo de HSH no identificados como gay y del grupo de HSH identificados como gay, no encontramos ninguna diferencia estadísticamente significativa en el uso de condón en la última relación sexual, en la presencia de síntomas de ITS en el último año, en el antecedente de pruebas de VIH previas y en la frecuencia de pruebas de VIH (Tabla 1 y 2). Tampoco hubo diferencias significativas en los planes de hacerse la prueba de VIH en los próximos 6 meses o 30 días (antes de haber sido expuestos a la intervención: Tabla 2).

Al comparar la historia de pruebas de VIH entre los participantes identificados como  gay y no identificados como gay, 135 (72.2%) de los participantes no identificados como gay y 159 (58.5%) de los participantes identificados como gay reportaron que nunca se habían hecho la prueba de VIH (p = 0.004).

Efectos de la Intervención

Después de una media de 125.5 días de observación (rango 42-209 días), 18 participantes identificados como gay y 11 participantes no identificados como gay acudieron a nuestra clínica por la prueba de VIH. No hubo una diferencia significativa en el reporte de intenciones de hacerse la prueba de VIH dentro de los próximos 6 meses entre los participantes que vieron el video y leyeron el texto del grupo de HSH identificados como gay (RR = 1.75; intervalo de confianza al 95% [IC 95%]: 0.77-3.97) y no identificados como gay (RR = 1.43, IC 95%: 0.87-2.36). Tampoco hubo una diferencia significativa en el reporte de intenciones de hacerse la prueba de VIH en los próximos 30 días (RR = 1.54, IC 95%: 0.74-3.20), en sacar una cita por Internet (RR = 1.11, IC 95%: 0.88-1.39) y en asistir a la clínica para hacerse la prueba de VIH (RR = 1.07, IC 95%: 0.40-2.85) entre los participantes que vieron el video y leyeron el texto del grupo de HSH identificados como gay. Sin embargo, los participantes del grupo de HSH no identificados como gay que vieron el video, reportaron con mayor frecuencia que tenían intenciones de hacerse la prueba de VIH en los próximos 30 días (RR = 2.77, IC 95%: 1.42-5.39), sacaron con mayor frecuencia una cita por Internet (RR = 1.48, IC 95%: 1.13 a 1.95) y acudieron con mayor frecuencia a nuestra clínica para hacerse la prueba de VIH (11.3% frente a 0%, p=0.001) en comparación con los participantes del grupo de HSH no identificados como gay que leyeron el texto (Tablas 3 y 4).

Entre los HSH no identificados como gay que acudieron a nuestra clínica, la mediana de días que tardaron en acudir desde que vieron el video hasta que vinieron a nuestra clínica fue de 3 días (rango: 0.5 - 60 días). Entre los HSH identificados como gay, la mediana de días que tardaron en acudir a nuestra clínica fue de 280 días (rango: 130 – 414) para los participantes que vieron el video y 255.5 días (rango: 123 – 432) para los que leyeron el texto (Figuras 2 y 3). En el grupo de HSH no identificados como gay que acudieron a nuestra clínica, 8 de los 11 participantes nunca se había hecho la prueba de VIH. En el grupo de HSH identificados como gay, 10 de un total de 18 participantes que asistieron a nuestra clínica nunca antes se habían hecho la prueba de VIH.

Aceptabilidad de la Intervención

En el grupo de HSH no identificados como gay, la aceptabilidad de la intervención fue similar entre los participantes que vieron el video y los que leyeron el texto (p=0.58). En el grupo de HSH identificados como gay, la aceptabilidad del video fue superior a la aceptabilidad del texto (p= 0.002; Tabla 5).

Servicios Clínicos de interés para los participantes

En el grupo de HSH no identificados como gay, los participantes que vieron el video estaban más interesados en recibir gratuitamente en nuestra clínica: Atención médica, consejería, preservativos y lubricantes, pruebas rápidas de VIH y sífilis (en comparación con los participantes que leyeron el texto).

En el grupo de HSH identificados como gay, los participantes que vieron el video estaban más interesados en la prueba rápida de VIH, en comparación con los participantes que leyeron el texto (Tabla 5).

Discusión

Los objetivos de este estudio fueron evaluar la eficacia de videos difundidos a través de Internet en lograr un cambio en el deseo de participantes HSH de hacerse la prueba de VIH dentro de los 6 meses y 30 días posteriores a la intervención, así como lograr que esta población se haga la prueba de VIH. Este último objetivo fue todo un reto si consideramos que la población que recibió nuestra intervención es una población marginada en el Perú que vive en un entorno donde ser HSH y tener VIH es altamente estigmatizante, y donde la disponibilidad de TARGA gratuito no siempre es conocida. Adicionalmente, nuestra población objetivo fue HSH que no se habían hecho la prueba de VIH durante el último año, lo que la define como una población más renuente a hacerse la prueba [5]. Es también importante considerar que no brindamos ni compensación monetaria por la participación, ni recursos adicionales para cubrir los gastos de transporte a nuestra clínica.

Los videos no fueron capaces de incrementar el porcentaje de participantes que planeaban hacerse la prueba de VIH dentro de los próximos 6 meses (paso del estadio de precontemplación al de contemplación) en ninguna de las dos poblaciones estudiadas. Como ocurre en otros estudios, el estadio de precontemplación es un estadio estable. En un estudio sobre riesgo de VIH en mujeres, se evaluó el estadio de cambio de las participantes con respecto al uso del condón en dos momentos distintos con un año de diferencia, y más del 50% de las participantes se mantuvo en el estadio de precontemplacion a través del estudio [23]. Según un estudio realizado por Prochaska et al., en el estadio de precontemplación, los participantes perciben que las desventajas de cambiar la conducta problema son superiores a las ventajas y el progreso del estadio de precontemplación a contemplación implica una mejora en la evaluación de las ventajas de cambiar [19]. Aunque nuestros videos resaltaron estas ventajas, la falta de cambio observado podría deberse al hecho de que 1) la intervención no se ajustaba a los temores o las razones específicas que cada participante tenía para no hacerse la prueba de VIH, sino a un grupo de razones para no hacerse pruebas identificadas en la investigación formativa, 2) el breve período de la intervención (la duración de los videos fue sólo cinco minutos), o 3) el breve período de tiempo desde el final de la intervención a la evaluación (minutos).

El video dirigido a los HSH no identificados como gay aumentó considerablemente el porcentaje de participantes que planeaban hacerse la prueba de VIH en los próximos 30 días. Este resultado demuestra que fuimos capaces de producir un cambio en los participantes del estadio de contemplación al de preparación. También logramos aumentar el porcentaje de participantes que sacaron una cita por Internet y que finalmente se hicieron la prueba de VIH en nuestra clínica (un cambio al estadio de acción). La mediana del tiempo para hacerse la prueba de VIH entre los participantes que acudieron a la clínica fue de sólo 3 días. Sin embargo, el video dirigido a los HSH identificados como gay no produjo dichos efectos. De hecho, entre los que asistieron a nuestra clínica, el tiempo medio para hacerse la prueba de VIH fue prolongado (280 días). Es probable que alguna otra razón, como un comportamiento de riesgo reciente, desencadenara la visita para hacerse la prueba de VIH.

Pueden haber varias explicaciones por las cuales el video dirigido a los HSH no identificados como gay produjo avances significativos en los estadios de cambio, mientras que el video dirigido a gays no lo hizo. La prueba de VIH fue nueva en general para el grupo de HSH no identificados como gay (72.2% de los participantes no identificados como gay frente a 58.5% de los participantes identificados como gay nunca se habían hecho la prueba de VIH), por lo que ellos estaban listos para que un poco de información los motive a hacerse la prueba. En contraste, el grupo de HSH identificados como gay ya habían considerado los pros y los contras de hacerse la prueba de VIH, por lo que la intervención en su conjunto, ya sea texto o video, no les añadió nada nuevo. El video dirigido a los HSH no identificados como gay, no muestra personas con resultados positivos a la prueba de VIH, a diferencia del video dirigido a los HSH identificados como gay. Esto pudo hacer menos evidente la posibilidad de obtener una prueba positiva, lo que se reconoce como la razón más común por la que los HSH no solicitan la prueba de VIH [24,25]. Otra razón podría ser que el mensaje de la intervención basada en texto no funcionó tan bien como el video para el grupo de HSH no identificados como  gay, pero sí funcionó para el grupo de HSH identificados como gay. Por último, existe la posibilidad de que el grupo de HSH no identificados como gay tenga una mayor receptividad a los videos que al texto en comparación con la población de HSH identificados como gay.

La aceptación del video y el texto fue elevada en este estudio (más del 80% consideraron estas intervenciones como «muy buenas» o «buenas»). En el grupo de HSH no identificados como gay los participantes que vieron el video estaban más interesados que los participantes que vieron el texto en recibir en nuestra clínica servicios tales como atención médica gratuita, consejería, condones y lubricantes, pruebas rápidas de VIH y sífilis. En cambio, en el grupo de HSH identificados como gay, los participantes que vieron el video estaban más interesados sólo en las pruebas rápidas de VIH en comparación con los participantes que leyeron el texto. Esto implica que el mensaje percibido del video en el grupo de HSH identificados como gay se centró en la prueba de VIH mientras que en el grupo de HSH no identificados como gay fue más diversificado a una variedad de opciones de cuidado de la salud. Este hallazgo también puede explicar por qué un número significativamente mayor de participantes no identificados como gay que vieron el video (en comparación con los que leyeron el texto) asistieron a la clínica.

Los datos empíricos en la literatura educativa o psicológica sugieren que el aprendizaje efectivo puede ser mejorado a través de presentaciones multimodales que combinan presentaciones auditivas-verbales y visuales [26]. Además, los videos en comparación con el texto son un medio visual que puede representar personajes con  los que los participantes pueden identificarse y también pueden proporcionar información y modelar estrategias exitosas para lograr una reducción del riesgo [27,28].

A nuestro entender este es el primer EA que ha abordado temas de VIH e ITS a través de Internet en un país en desarrollo. En países desarrollados donde el medio de acceso a Internet es predominantemente en el domicilio (versus cabinas públicas) y la velocidad de la conexión es mayor [7], se han realizado EA anteriores, algunos ejemplos son: La evaluación de una red informática basada en el hogar para reducir el aislamiento social de las personas con VIH [29]; la evaluación de un sistema de recordatorio mediante el uso de localizadores personales para mejorar la adherencia a antirretrovirales [30]; la evaluación del efecto de intervenciones en Internet específicas y no específicas a las necesidades del participante para aumentar el uso de condones e incrementar el número de HSH que se hacen pruebas de ITS y VIH [31], y la evaluación de la aceptabilidad y la eficacia de intervenciones de reducción de riesgo de VIH realizadas a través de Internet entre HSH de zonas rurales [10]. La factibilidad de realizar el estudio que les hemos presentado en un entorno con recursos limitados y el uso de pruebas de VIH como una medida real de la eficacia de la intervención, abre nuevas posibilidades no sólo para seguir estudiando y perfeccionando nuevas intervenciones, sino para extender estas a otras poblaciones y enfermedades.

Un hallazgo interesante fue el elevado número de participantes que sacaron una cita por Internet y que finalmente no vinieron. Algunas posibles explicaciones para esto son que la Internet es atractiva para los HSH debido al anonimato que ofrece, y funcionará principalmente cuando los participantes estén en línea, pero no si les pedimos que acudan en persona. La accesibilidad a la clínica puede ser otro problema. Lima es una ciudad de 53 kilómetros de largo con 8 millones de habitantes que se movilizan principalmente a través de un sistema de transporte público lento. Esto hace difícil que los participantes acudan a nuestra única clínica ubicada en el centro de Lima. Por otra parte, no hemos incluido recordatorios de la citas, ya que este estudio fue diseñado específicamente para evaluar el efecto de un video sobre un texto. En estudios futuros, la implementación de mensajes de seguimiento y de recordatorios personalizados que refuercen las intervenciones pueden aumentar el número de participantes que finalmente acuden a la clínica. Además, hemos desarrollado videos de 5 minutos de duración que tenían por intención abordar colectivamente todos los motivos que los participantes tenían para no hacerse la prueba de VIH; este video puede no haberse ajustado suficientemente a los motivos específicos que cada participante tenía para no hacerse la prueba de VIH.

Nuestro estudio tiene algunas limitaciones. En primer lugar, la muestra no es representativa de la población de HSH de Lima, o de toda la población de HSH que visitan las páginas Web gay peruanas. En este estudio, hemos tratado de llegar a los HSH de mayor riesgo con acceso a Internet. En segundo lugar, es probable que nuestro muestreo este sesgado en términos de nivel educativo y edad. En tercer lugar, no hemos sido capaces de recopilar datos sobre los participantes que pudieron haber asistido a otras clínicas para ser tamizados para VIH. En cuarto lugar, es posible que tengamos errores de auto-representación de algunos participantes (por ejemplo, participantes mujeres respondiendo como varones), esto produce errores de clasificación que pueden disminuir la validez interna de nuestro estudio. Finalmente, no hemos sido capaces de evaluar en qué medida los participantes fueron expuestos a las intervenciones, es decir, si los participantes realmente leyeron el texto o vieron el video.

La Internet se ha convertido en un método alternativo que puede usarse para involucrar poblaciones de alto riesgo y de recursos limitados en intervenciones de prevención del VIH orientadas a brindar un diagnóstico y tratamiento más temprano. Las futuras intervenciones a través de Internet en el Perú deberían centrarse en sub-poblaciones más específicas dentro de los grupos de HSH, y deberían ser adaptadas específicamente a las razones más importantes que los participantes tienen para no hacerse la prueba de VIH. La duración del seguimiento se debe basar en el tiempo que toman los participantes para avanzar de un estadio de cambio al siguiente. La intervención también debería incluir una amplia variedad de lugares donde los participantes puedan hacerse las pruebas de VIH.

**Agradecimientos:**

Los autores desean agradecer a todos los miembros del proyecto SOMOS de Vía Libre por su apoyo en el desarrollo de este proyecto.

Referencias

1. UNAIDS (2007) *AIDS Epidemic Update.* Geneva: Joint United Nations Programme on HIV/AIDS Available: http://data.unaids.org/pub/EPISlides/2007/2007_epiupdate_en.pdf. Accessed 2010 March 10.

2. Orellana ER, Picciano JF, Roffman RA, Swanson F, Kalichman SC (2006) Correlates of nonparticipation in an HIV prevention program for MSM. AIDS Educ Prev 18(4): 348-61.

3. Kusunoki L, Guanira J, Navarro C, Velasquez C (2005) Report of Monitoring the declaration of commitment on HIV/AIDS 2005. Available: http://data.unaids.org/pub/Report/2006/2006_country_progress_report_peru_en.pdf. Accessed 2010 March 10.

4. Peru Country Progress Report UNGASS (2008). Available: http://data.unaids.org/pub/Report/2008/peru_2008_country_progress_report_sp_es.pdf. Accessed 2010 March 10.

5. Blas M (2007) *Web-based survey to assess risk behaviors for sexually transmitted infections and HIV among men who have sex with men from Peru*. Master of Public Health Thesis. University of Washington.

6. Blas MM, Alva IE, Cabello R, Garcia PJ, Carcamo C, et al. (2007) Internet as a tool to access high-risk men who have sex with men from a resource-constrained setting: a study from Peru. Sex Transm Infect 83(7): 567-570.

7. Curioso WH, Blas MM, Nodell B, Alva IE, Kurth AE (2007) Opportunities for providing web-based interventions to prevent sexually transmitted infections in Peru. PLoS Med 4(2): e11.

8. Bull SS, McFarlane M, King D (2001) Barriers to STD/HIV prevention on the Internet. Health Educ Res 16(6): 661-70.

9. Levine DK, Scott KC, Klausner JD (2005) Online syphilis testing--confidential and convenient. Sex Transm Dis 32(2): 139-41.

10. Bowen AM, Horvath K, Williams ML (2007) A randomized control trial of Internet-delivered HIV prevention targeting rural MSM. Health Educ Res 22(1): 120-7.

11. Centers for Disease Control and Prevention (CDC) (2004) Using the Internet for partner notification of sexually transmitted diseases. Los Angeles County, California, 2003. MMWR Morb Mortal Wkly Rep 53(6): 129-31.

12. Ross MW (2002) The Internet as a medium for HIV prevention and counseling. Focus 17(5): 4-6.

13. Warner L, Klausner JD, Rietmeijer CA, Malotte CK, O'Donnell L, et al. (2008) Effect of a brief video intervention on incident infection among patients attending sexually transmitted disease clinics. PLoS Med 5(5): e135.

14. (2008) Online film successful in reducing certain HIV risk behaviors. At-risk people watch 9-minute video. AIDS Alert 23(2):18-9.

15. Nijland N, van Gemert-Pijnen J, Boer H, Steehouder MF, Seydel ER (2008) Evaluation of internet-based technology for supporting self-care: problems encountered by patients and caregivers when using self-care applications. J Med Internet Res 10(2): e13.

16 LimeSurvey, the open source survey application. Available: http://www.limesurvey.org. Accessed 2008 June 20.

17. Get tested with Via Libre (for non-gay identified MSM). Available: http://www.youtube.com/watch?v=mKeR9-YVswg. Accessed 2010 March 15.

18. Get tested with Via Libre (for gay-identified MSM). Available: http://www.youtube.com/watch?v=QN4BmLopzG0. Accessed 2010 March 15.

19. Get tested with Via Libre (for trans). Available: http://www.youtube.com/watch?v=nT131RlToeo. Accessed 2010 March 15.

20. El Gremio, centro comunitario de Lucha Contra el SIDA. Campana Hazte la Prueba. Available: http://www.gremiosanluis.com/sexoseguro_prueba.htm. Accessed 2007 July 20.

21. DiClemente CC, Prochaska JO, Fairhurst SK, Velicer WF, Velasquez MM et al. (1991) The process of smoking cessation: an analysis of precontemplation, contemplation, and preparation stages of change. J Consult Clin Psychol 59(2): 295-304.

22. Prochaska JO, Velicer WF, Rossi JS, Goldstein MG, Marcus BH, et al. (1994) Stages of change and decisional balance for 12 problem behaviors. Health Psychol 13(1): 39-46.

23. Evers KE, Harlow LL, Redding CA, LaForge RG (1998) Longitudinal changes in stages of change for condom use in women. Am J Health Promot 13(1): 19-25.

24. Kellerman SE, Lehman JS, Lansky A, Stevens MR, Hecht FM, et al. (2002) HIV testing within at-risk populations in the United States and the reasons for seeking or avoiding HIV testing. J Acquir Immune Defic Syndr 31(2): 202-10.

25. Mikolajczak J, Hospers HJ, Kok G (2006) Reasons for not taking an HIV-test among untested men who have sex with men: an Internet study. AIDS Behav 10(4): 431-5.

26. Mayer RE (2001) *Multimedia Learning.* New York: Cambridge University Press. 222 p.

27. Myint-U A, Bull S, Greenwood GL, Patterson J, Rietmeijer CA, et al. (2008) Safe in the City: Developing an Effective Video-Based Intervention for STD Clinic Waiting Rooms. Health Promot Pract doi:10.1177/1524839908318830

28. O'Donnell L, San Doval A, Duran R, O'Donnell CR (1995) The effectiveness of video-based interventions in promoting condom acquisition among STD clinic patients. Sex Transm Dis 22(2): 97-103.

29. Flatley-Brennan P (1998) Computer network home care demonstration: a randomized trial in persons living with AIDS. Comput Biol Med 28(5): 489-508.

30. Safren SA, Hendriksen ES, Desousa N, Boswell SL, Mayer KH (2003) Use of an on-line pager system to increase adherence to antiretroviral medications. AIDS Care 15(6): 787-93.

31. Bull SS, Lloyd L, Rietmeijer C, McFarlane M (2004) Recruitment and retention of an online sample for an HIV prevention intervention targeting men who have sex with men: the Smart Sex Quest Project. AIDS Care 16(8): 931-43.

**Figura 1: Proceso de aleatorización**

**Figura 2: Porcentaje de HSH no identificados como gay que se hicieron la prueba de VIH según tiempo desde la aleatorización (en días).**

**Figura 3: Porcentaje de HSH identificados como  gay que se hicieron la prueba de VIH según  tiempo desde la aleatorización (en días).**

**Tabla 1: Comparación de las características demográficas, el acceso a la información sobre VIH, las conductas de riesgo para ITS y síntomas de ITS según grupo de intervención en HSH identificados y no identificados como gay.**

|  | **HSH no identificados como gay** | | | **HSH identificados como gay** | | |
| --- | --- | --- | --- | --- | --- | --- |
|  | **Video** | **Texto** | **P** | **Video** | **Texto** | **P** |
|  | **N(%)** | **N(%)** |  | **N(%)** | **N(%)** |  |
| **Edad (rango)** | 26.4 (18-50) | 26.2 (18-54) | 0.84 | 26.9  (18-52) | 25.0 (18-61) | 0.04 |
| **Educación** |  |  | 0.45 |  |  | 0.38 |
| Secundaria incompleta o menos | 6 (6.3) | 2 (2.2) |  | 3 (2.1) | 5 (3.9) |  |
| Secundaria completa | 13 (13.5) | 13 (14.4) |  | 18 (12.8) | 22 (17.3) |  |
| Universidad/Instituto incompleto | 33 (34.4) | 38 (42.2) |  | 55 (39.0) | 53 (41.7) |  |
| Universidad/Instituto completo | 44 (45.8) | 37 (41.1) |  | 65 (46.1) | 47 (37.0) |  |
| **Lugar de acceso a Internet** |  |  | 0.41 |  |  | 0.53 |
| Cabina Pública | 48 (50.5) | 40 (44.4) |  | 60 (43.2) | 50 (39.4) |  |
| Casa/Trabajo | 47 (49.5) | 50 (55.6) |  | 79 (56.8) | 77 (60.6) |  |
| **Orientación Sexual** |  |  | 0.19 |  |  | 0.05 |
| Homosexual | 21 (21.6) | 27 (30.0) |  | 130 (91.5) | 127 (97.7) |  |
| Bisexual | 76 (78.4) | 63 (70.0) |  | 12 (8.5) | 3 (2.3) |  |
| **Auto-identificación** |  |  | 0.25 |  |  | 0.72 |
| Bisexual | 74 (76.3) | 78 (86.7) |  | --- | --- |  |
| Heterosexual | 8 (8.3) | 6 (6.7) |  | --- | --- |  |
| Hombre | 13 (13.4) | 6 (6.7) |  | --- | --- |  |
| Flete | 2 (2.1) | 0 (0.0) |  | --- | --- |  |
| Gay | --- | --- |  | 123 (86.6) | 115 (88.5) |  |
| Caleta | --- | --- |  | 19 (13.4) | 15 (11.5) |  |
| **Rol Sexual** |  |  | 0.45 |  |  | 0.48 |
| Insertivo | 38 (39.6) | 27 (31.0) |  | 11 (7.9) | 16 (12.5) |  |
| Receptivo | 10 (10.4) | 12 (13.8) |  | 45 (32.4) | 39 (30.5) |  |
| Versátil | 48 (50) | 48 (55.2) |  | 83 (59.7) | 73 (57.0) |  |
| **Recibió información sobre HIV en el último año** | 31 (33) | 33 (37.1) | 0.64 | 54 (39.1) | 48 (37.8) | 0.90 |
| **Usó condón en la última relación sexual** | 47 (50.0) | 44 (53.0) | 0.88 | 60 (43.5) | 64 (52.9) | 0.26 |
| **Síntomas de ITS** |  |  |  |  |  |  |
| Disuria | 18 (18.6) | 17 (18.9) | 1.00 | 19 (13.4) | 16 (12.3) | 0.86 |
| Descarga  uretral | 6 (6.2) | 7 (7.8) | 0.78 | 8 (5.6) | 8 (6.2) | 1.00 |
| Descarga anal | 1 (1) | 1 (1.1) | 1.00 | 0 (0.0) | 2 (1.5) | 0.23 |
| Ulceras en el pene | 5 (5.2) | 4 (4.4) | 1.00 | 8 (5.6) | 8 (6.2) | 1.00 |
| Ulceras en el ano | 7 (7.2) | 4 (4.4) | 0.54 | 4 (2.8) | 9 (6.9) | 0.16 |
| Verrugas en el pene | 4 (4.1) | 0 (0) | 0.12 | 6 (4.2) | 1 (0.8) | 0.12 |
| Verrugas en el ano | 5 (5.2) | 2 (2.2) | 0.45 | 8 (5.6) | 5 (3.9) | 0.58 |

**Tabla 2: Comparación de la historia de pruebas de VIH y los planes para hacerse la prueba de VIH según el grupo de intervención entre HSH identificados y no identificados como gay.**

|  | **HSH no identificados como gay** | | | **HSH identificados como gay** | | |
| --- | --- | --- | --- | --- | --- | --- |
|  | **Video** | **Texto** | **P** | **Video** | **Texto** | **P** |
|  | **N(%)** | **N(%)** |  | **N(%)** | **N(%)** |  |
| **Se ha hecho pruebas de VIH antes*** |  |  | 0.63 |  |  | 0.24 |
| Nunca | 73 (75.3) | 62 (68.9) |  | 76 (53.5) | 83 (63.9) |  |
| Hace 1-2 años | 15 (15.5) | 14 (15.6) |  | 38 (26.8) | 30 (23.1) |  |
| Hace 2-5 años | 5 (5.2) | 8 (8.9) |  | 23 (16.2) | 12 (9.2) |  |
| Hace más de 5 años | 4 (4.1) | 6 (6.7) |  | 5 (3.5) | 5 (3.9) |  |
| **Frecuencia de pruebas de VIH** |  |  | 0.82 |  |  | 0.80 |
| Sólo una vez | 14 (66.7) | 19 (76.0) |  | 29 (48.3) | 28 (62.2) |  |
| Dos veces al año |  |  |  | 1 (1.7) | 0 (0.0) |  |
| Cada año | 2 (9.5) | 3 (12.0) |  | 14 (23.3) | 8 (17.8) |  |
| Cada 2 -3 años | 3 (14.3) | 2 (8.0) |  | 11 (18.3) | 7 (15.6) |  |
| Cada 4-5 años | 0 (0.0) | 0 (0.0) |  | 3 (5.0) | 1 (2.2) |  |
| Otro | 2 (9.5) | 1 (4.0) |  | 2 (3.3) | 1 (2.2) |  |
| **Está planeando hacerse pruebas de VIH  en los próximos  6 meses (antes de ser expuesto  a la  intervención)** | 60 (66.0) | 54 (63.5) | 0.76 | 96 (73.9) | 86 (71.7) | 0.78 |
| **Está planeando hacerse pruebas de VIH  en los próximos  30 días (antes de ser expuesto  a la  intervención)** | 32 (36.4) | 19 (24.1) | 0.10 | 48 (38.1) | 38 (33.9) | 0.59 |

* Los participantes que se hicieron pruebas de VIH hace 12 meses o menos (estadio de acción) no fueron aleatorizados

**Tabla 3: Efectos de la intervención en el grupo de HSH no identificados como gay.**

|  | **Intervención** | |  |
| --- | --- | --- | --- |
|  | **Video** | **Texto** |  |
|  | **N (%)** | **N (%)** | **Razón de Riesgo^c^**  **(IC 95%)** |
| **Está planeando hacerse la prueba de VIH  en los próximos  6 meses después de  haberse expuesto a la intervención^a^** |  |  |  |
| Sí | 18 (62.1) | 13 (43.3) | 1.43 (0.87-2.36) |
| No | 11 (37.9) | 17 (56.7) | Ref |
| **Está planeando hacerse la prueba de VIH  en los próximos  30 días después de  haberse expuesto a la intervención^b^** |  |  |  |
| Sí | 15 (62.5) | 4 (15.4) | 2.77 (1.42-5.39) |
| No | 9 (37.5) | 22 (84.6) | Ref |
| **Sacó una cita por  Internet** |  |  |  |
| Sí | 64 (66.0) | 40 (44.4) | 1.48 (1.13 - 1.95) |
| No | 33 (34.0) | 50 (55.6) |  |
| **Acudió a la clínica** |  |  |  |
| Sí | 11 (11.3) | 0 (0.0) | ...^d^ |
| No | 86 (88.7) | 90 (100) | Ref |

^a^ Restringido a los que antes de exponerse a la intervención, no planeaban hacerse la prueba de VIH dentro de los siguientes 6 meses

^b^ Restringido a los que antes de exponerse a la intervención, planeaban hacerse la prueba de VIH dentro de los siguientes 6 meses pero no planeaban hacerse la prueba en los próximos  30 días.

^c^ La Razón de Riesgo para planear hacerse la prueba de VIH en los próximos 6 meses y los próximos  30 días fue calculada usando Mantel Haenszel y la Razón de Riesgo para acudir a la clínica fue calculada usando  regresión de riesgos proporcionales de Cox.

^d^ Hazard Ratio no puede ser estimado.

**Tabla 4: Efectos de la intervención en el grupo de HSH identificados como gay.**

|  | **Intervención** | |  |  |
| --- | --- | --- | --- | --- |
|  | **Video** | **Texto** | **Razón de Riesgo^c^ (IC 95%)** | |
|  | **N (%)** | **N (%)** | **Crudo** **(IC 95%)** | **Ajustado (IC 95%)^d^** |
| **Está planeando hacerse la prueba de VIH en los próximos  6 meses después de  haberse expuesto a la intervención ^a^** |  |  |  |  |
| Sí | 17 (58.6) | 11 (35.5) | 1.65 (0.94-2.91) | 1.75 ( 0.77-3.97) |
| No | 12 (41.4) | 20 (64.5) |  |  |
| **Está planeando hacerse la prueba de VIH en los próximos 30 días después de haberse expuesto a la intervención^b^** |  |  |  |  |
| Sí | 19 (50.0) | 8 (21.6) | 2.31 (1.16-4.61) | 1.54 (0.74-3.20) |
| No | 19 (50.0) | 29 (78.4) |  |  |
| **Sacó una cita por  Internet** |  |  |  |  |
| Sí | 84 (59.2) | 71 (54.6) | 1.08 (0.88-1.33) | 1.11 (0.88-1.39) |
| No | 58 (40.9) | 59 (45.4) |  |  |
| **Acudió a la clínica** |  |  |  |  |
| Sí | 8 (5.6) | 10 (7.7) | 1.03 (0.39-2.70) | 1.07 (0.40-2.85) |
| No | 134 (94.4) | 120 (92.3) |  |  |

^a^ Restringido a los que antes de exponerse a la intervención, no planeaban hacerse la prueba de VIH dentro de los siguientes 6 meses.

^b^ Restringido a los que antes de exponerse a la intervención, planeaban hacerse la prueba de VIH dentro de los siguientes 6 meses pero no planeaban hacerse la prueba de VIH en los próximos 30 días.

**^c^** La Razón de Riesgo para planear hacerse la prueba de VIH en los próximos 6 meses y los próximos  30 días fue calculada usando Mantel Haenszel y la Razón de Riesgo para acudir a la clínica fue calculada usando  regresión de riesgos proporcionales de Cox.

**^d^** Ajustada por edad y orientación sexual

**Tabla 5: Aceptabilidad del video y texto y servicios que los participantes del grupo de HSH identificados y no identificados como gay estaban interesados en recibir en la clínica.**

|  | **HSH no identificados como gay** | | | **HSH identificados como gay** | | |
| --- | --- | --- | --- | --- | --- | --- |
|  | **Video** | **Texto** | **P** | **Video** | **Texto** | **P** |
|  | **N(%)** | **N(%)** |  | **N(%)** | **N(%)** |  |
| **Calificación de la intervención** |  |  | 0.58 |  |  | 0.002 |
| Muy buena | 55 (60.0) | 44 (50.0) |  | 93 (66.9) | 61 (47.7) |  |
| Buena | 30 (32.6) | 34 (38.6) |  | 39 (28.1) | 45 (35.2) |  |
| Regular | 6 (6.5) | 9 (10.2) |  | 7 (5.0) | 15 (11.7) |  |
| Mala | 0 | 0 |  | 0 | 5 (3.9) |  |
| Muy mala | 1 (1.1) | 1 (1.1) |  | 0 | 2 (1.6) |  |
| **Servicios que los participantes estaban interesados en recibir en la clínica** |  |  |  |  |  |  |
| Atención médica | 56 (57.7) | 34 (37.8) | 0.008 | 84 (59.2) | 65 (50.0) | 0.14 |
| Consejería | 58 (59.8) | 32 (35.6) | 0.001 | 77 (54.2) | 63 (48.5) | 0.40 |
| Condones y lubricantes | 48 (49.5) | 31 (34.4) | 0.04 | 70 (49.3) | 61 (46.9) | 0.72 |
| Prueba rápida de VIH | 63 (65.0) | 42 (46.7) | 0.01 | 94 (66.2) | 60 (46.2) | 0.001 |
| ELISA para VIH | 37 (38.1) | 27 (30.0) | 0.28 | 39 (27.5) | 43 (33.1) | 0.36 |
| Prueba de sífilis | 42 (43.3) | 25 (27.8) | 0.03 | 55 (38.7) | 45 (34.6) | 0.53 |
